# A detailed analysis of innate and adaptive immune responsiveness upon infection with *Salmonella enterica* serotype Enteritidis in young broiler chickens

**DOI:** 10.1186/s13567-021-00978-y

**Published:** 2021-08-17

**Authors:** Nathalie Meijerink, Robin H. G. A. van den Biggelaar, Daphne A. van Haarlem, J. Arjan Stegeman, Victor P. M. G. Rutten, Christine A. Jansen

**Affiliations:** 1 Department Biomolecular Health Sciences, Division Infectious Diseases and Immunology, Faculty of Veterinary Medicine, Utrecht University, Utrecht, The Netherlands; 2 Department Population Health Sciences, Division Farm Animal Health, Faculty of Veterinary Medicine, Utrecht University, Utrecht, The Netherlands; 3 Department of Veterinary Tropical Diseases, Faculty of Veterinary Science, University of Pretoria, Pretoria, South Africa; 4 Present Address: Department of Animal Sciences, Cell Biology and Immunology Group, Wageningen University and Research, Wageningen, The Netherlands

**Keywords:** Broiler chickens, *Salmonella enterica* serotype Enteritidis, Innate, Adaptive, Immunity, NK cells, Antigen-presenting cells, T cells, IELs

## Abstract

*Salmonella enterica* serotype Enteritidis (SE) is a zoonotic pathogen which causes foodborne diseases in humans as well as severe disease symptoms in young chickens. More insight in innate and adaptive immune responses of chickens to SE infection is needed to understand elimination of SE. Seven-day-old broiler chickens were experimentally challenged with SE and numbers and responsiveness of innate and adaptive immune cells as well as antibody titers were assessed. SE was observed in the ileum and spleen of SE-infected chickens at 7 days post-infection (dpi). At 1 dpi numbers of intraepithelial cytotoxic CD8^+^ T cells were significantly increased alongside numerically increased intraepithelial IL-2Rα^+^ and 20E5^+^ natural killer (NK) cells at 1 and 3 dpi. At both time points, activation of intraepithelial and splenic NK cells was significantly enhanced. At 7 dpi in the spleen, presence of macrophages and expression of activation markers on dendritic cells were significantly increased. At 21 dpi, SE-induced proliferation of splenic CD4^+^ and CD8^+^ T cells was observed and SE-specific antibodies were detected in sera of all SE-infected chickens. In conclusion, SE results in enhanced numbers and activation of innate cells and we hypothesized that in concert with subsequent specific T cell and antibody responses, reduction of SE is achieved. A better understanding of innate and adaptive immune responses important in the elimination of SE will aid in developing immune-modulation strategies, which may increase resistance to SE in young broiler chickens.

**Supplementary Information:**

The online version contains supplementary material available at 10.1186/s13567-021-00978-y.

## Introduction

*Salmonella enterica* serotype Enteritidis (SE) is one of the leading causes of foodborne diseases in humans, most often due to poultry products that are not well prepared. In chickens infected with faecal *Salmonellae* via oral or respiratory routes, SE colonizes the intestinal tract and disseminates systemically to tissues such as the liver and spleen [1, 2]. In young chickens it can lead to severe disease and death, whereas adult chickens are often subclinically infected with SE, carrying the bacteria in their intestines [3]. Prevention of SE infection in poultry is thus important for health and welfare of young chickens and to avoid substantial economic production losses in the poultry sector as a consequence. In addition, SE prevention in poultry is significant for the health and wellbeing of humans as well as to avoid loss of productivity and health care costs. Since therapeutic treatment of SE infection in chickens with antibiotics is not advised due to limited effectiveness and risk of antibiotic resistance, the use of immune-modulatory strategies to increase the resistance to SE is encouraged [4]. More insight in innate and adaptive immune responses and their interaction in response to SE infection in young broiler chickens will facilitate the design of these strategies.

In young chickens, immunity largely depends on maternal antibodies as well activity of the innate immune system, with natural killer (NK) cells and macrophages as key players [5, 6]. Due to the low numbers of NK cells that can be isolated from the caecum [7, 8] and since the ileum is generally considered as a site of immune activation with many lymphoid structures [9, 10], we set out to study immune responses induced by SE in the ileum. NK cells are particularly abundant amongst intestinal intraepithelial lymphocytes (IELs) [11, 12], which are also rich in γδ T cells and CD8^+^ T cells expressing the αβ T cell receptor (TCR) [11, 13]. Macrophages, dendritic cells (DCs) and CD4^+^ T cells are located directly underneath the intestinal epithelium [14]. The adaptive immune system is not fully developed yet upon hatch and functional T- and B-cell responses are observed after approximately 2 to 3 weeks of life [5, 6].

The early response of the innate immune system in chickens within 1 week post-SE infection is characterized by the upregulation of genes associated with “defense/pathogen response” [15], inflammation [16–18], NK cell-mediated cytotoxicity [19] and production and secretion of the cytokine IFNγ [20]. Other studies have shown the influx of heterophils and macrophages in the spleen and γδ T cells in the caecum, and expression of activation-related genes in the respective cell types during the first response to SE in chickens [21, 22]. However, the effect of SE infection on the function of NK cells in chickens has not been studied so far. In chickens, intraepithelial NK cells comprise a major CD3^−^ IL-2Rα^+^ subset [11, 12] and a minor CD3^−^ 20E5^+^ subset [12], both having cytotoxic capacity but to different degrees. In mice and humans, high cytotoxic activity [23, 24] and IFN-γ production [24, 25] by NK cells have been shown to result in resistance to *Salmonella enterica* serotype Typhimurium. This suggests an important role for NK cells as well in the first response to SE.

The development of T and B cell responses is initiated with the activation of professional antigen presenting cells (APCs) such as DCs and macrophages. The presence [26] and activity of intestinal macrophages [27–29], identified by the expression of mannose receptor C-type 1-like B (MRC1LB) [30], and bone marrow-derived DCs [31] increases during SE infection. Furthermore, oral infection of 1-day-old specific-pathogen-free chickens with SE elicits increased mRNA expression of chemokines and macrophages are attracted to the ileum within 24 h [32]. In addition, it has been shown that decreased activity of peritoneal macrophage is associated with increased susceptibility for systemic dissemination of SE in chickens [33]. On the other hand, *Salmonella* species have been found to resist killing by macrophages in mammals [34] and chickens [35, 36] and even use macrophages as a carrier for systemic dissemination [37]. Despite their involvement in SE infection, a detailed analysis of the effect of SE infection on the function of APCs in chickens has not been performed to date.

Initial T cell responsiveness to SE in chickens has been observed within 1 week, including increased presence of γδ T cells in the intestine, blood and spleen after vaccination with live-attenuated SE or SE infection as compared to SE negative chickens [20, 22]. In addition, enhanced mRNA expression of cytotoxic activity-related genes was observed in the spleen of SE-infected compared to uninfected chickens [38]. Two and three weeks post-vaccination or infection, a second increase in presence of γδ T cells was observed in the spleen and intestine, respectively, compared to non-immunized chickens [22]. CD4^+^ helper T cells and CD8^+^ cytotoxic T cells were also shown to increase in presence within 1 week post-SE infection and vaccination in the intestine, as compared to uninfected and non-vaccinated chickens [20, 26, 27]. CD8^+^ cytotoxic T cells kill infected host cells, while CD4^+^ helper T cells release cytokines like IL-2 and IFNγ to further stimulate NK cells, and macrophages and CD8^+^ cytotoxic T cells respectively, and promote the differentiation of B cells into antibody-producing plasma cells. Antibody responses involved in elimination of SE partly depend on maternal antibodies and on the production of IgA in the intestine [39, 40], as well as IgM, IgA and IgY antibodies in blood [40, 41]. Whereas previous studies have focussed on specific aspects of the immune responses, the present study combines cellular assays to analyze both the innate and adaptive immune responses in the IEL population and spleen upon SE infection in chickens.

In this study, we investigated how *Salmonella enterica* serotype Enteritidis infection in young broiler chickens affects presence and activation of innate and adaptive immune cells in the IEL population and spleen to obtain more insight in the contribution of the immune system to elimination of the infection. Numbers of SE in ileum and spleen were determined alongside differences in kinetics of presence and activation status of NK cell and APC subsets between uninfected and infected chickens. The subsequent adaptive responses were determined including presence and activation of γδ T cell, CD4^+^ and CD8^+^ T cell subsets, and serum antibody levels. Hence the present study provides an extensive overview of intraepithelial and systemic immune responses that are evoked by SE infection in young broiler chickens. Based on the phenotypical and functional data obtained, we will hypothesize on how the various elements of the immune system interact and contribute to elimination of the SE infection, and on potential strengthening of immune responsiveness by immunomodulation strategies, which may prevent SE infection and colonization, and thus increase chicken health and welfare as well as safety of food of chicken origin.

## Materials and methods

### Animals and husbandry

A total of 30 respectively 35 Ross 308 17- and 18-day old embryonated eggs were obtained from the same parent flock of a commercial hatchery (Lagerwey, the Netherlands). Eggs were disinfected with 3% hydrogen peroxide and placed in disinfected hatchers in two different stables (ED17 eggs: uninfected chickens and ED18 eggs: SE-infected chickens) at the facilities of the Department of Population Health Sciences, Faculty of Veterinary Medicine, Utrecht University, the Netherlands. Cleaning the eggs with a low concentration of hydrogen peroxide is a standard procedure. It is highly unlikely that it influences intestinal microbiota composition as described in a previous study [42]. Directly upon hatch, chickens were weighed, labelled and female and male chickens were equally distributed in floor pens of 2 × 2 m lined with wood shavings (2 kg/m^2^), and received water and standard *Salmonella*-free commercial starter and grower feeds ad libitum (Research Diet Services, the Netherlands). A standard lighting and temperature scheme for Ross broiler chickens was used for both stables.

The animal experiment was approved by the Dutch Central Authority for Scientific Procedures on Animals and the Animal Experiments Committee (registration number AVD1080020174425) of Utrecht University (the Netherlands) and all procedures were done in full compliance with all relevant legislation.

### Experimental design

Before the start of the experiment at day 3, five chickens per group [uninfected (*n* = 30) and SE-infected (*n* = 35)] were randomly selected and sacrificed for collection of ileum (± 10 cm distal from Meckel’s diverticulum) and spleen to confirm absence of SE before inoculation. At day 7 [0 days post-infection (dpi)], five chickens of the SE-infected group only, were randomly selected and sacrificed for collection of ileum and spleen, to determine baseline levels of the various immune parameters as well as absence of SE before infection. Subsequently, chickens of the SE-infected group were challenged at day 7 (0 dpi) by oral inoculation of 0.25 mL brain heart infusion (BHI) medium containing 1.12 × 10^6^ colony-forming units (CFUs) SE, whereas chickens in the other stable (uninfected) were inoculated with 0.25 mL BHI medium. At days 8 (1 dpi), 10 (3 dpi), 14 (7 dpi), 21 (14 dpi) and 28 (21 dpi), five chickens per group were randomly selected and sacrificed for collection of ileum and spleen to determine bacterial CFUs as well as numbers and function of NK cells and T cells. At days 7, 8, 10 and 14 also spleen APCs were assessed. At days 7, 14, 21 and 28 blood (at least 5 mL) was collected in EDTA tubes (VACUETTE® K3E EDTA, Greiner Bio-One, the Netherlands) for determination of SE-specific antibody levels. At day 28, splenic lymphocytes were also used to assess SE-induced T cell reactivity in a proliferation assay. The use of five chickens per group per time point was calculated using power analysis (Sample size & power calculator, LASEC, China). All chickens were weighed prior to post-mortem analyses to determine the growth curve. To calculate absolute cell numbers, ileum segments and spleens were weighed immediately after collection of the tissues, prior to isolation of cells. After isolation, cell numbers in the resulting suspension were calculated. This resulted in the total cell number, expressed as IELs per mg ileum or leukocytes per mg spleen. To calculate the absolute numbers of NK cells, APCs and T cells within the live IEL or leukocyte populations, the percentages of cells positive for the markers expressed on these cell types were used which were determined in the flow cytometry analyses. Absolute cell numbers were calculated using the following formula: (absolute number IELs or leukocytes per mg tissue) × (percentage positive cells in the gate of interest of the live lymphocyte or leukocyte population).

### SE culture

The *Salmonella enterica* serotype Enteritidis strain (K285/93 Nal^res^) was kindly provided by Dr E. Broens, director of the Veterinary Microbiological Diagnostic Center (VMDC) of the Faculty of Veterinary Medicine, Utrecht University, and cultured as described previously [43]. In short, from an overnight culture of the SE strain on blood agar (Oxoid, the Netherlands) a single colony was used to inoculate 45 mL BHI medium (Oxoid), which was incubated aerobically overnight at 200 rpm in a shaking incubator (Certomat BS-1, B. Braun Biotech international, Sweden) at 37 °C. The OD value of a sample of the SE culture diluted 1:10 in PBS was measured using a Ultrospec 2000 (Pharmacia Biotech, Sweden), the SE concentration was calculated from a previously determined growth curve, and SE were diluted in BHI medium to 4 × 10^6^ CFU/mL, to constitute the inoculum. The exact SE concentration of the inoculum, determined by counting the number of CFUs of plated serial dilutions after overnight culture, was 4.49 × 10^6^ CFU/mL.

For the T cell proliferation assay, SE was fixed by resuspending 3.8 × 10^9^ CFUs in 100 µL PBS with 1% formaldehyde (Sigma-Aldrich, the Netherlands) and incubation for 5 min at RT, while the suspension was vortexed shortly every minute. After fixation, the bacteria were washed four times in 1 mL PBS by centrifugation at 15 000 × *g* to remove the supernatants (Heraeus Pico 17 Centrifuge, Thermo Fisher Scientific). Finally, the bacteria were resuspended in 380 µL X-VIVO 15 cell culture medium (Lonza, the Netherlands) with 50 µg/mL gentamycin (Gibco™, the Netherlands) to create a concentration of 10^7^ CFU/mL and stored at 4 °C until further use.

### Isolation of cells

The procedures to isolate IELs from ileum and leukocytes from spleen were performed as described previously [12, 44]. Ileum segments were washed with PBS to remove the contents, cut into sections of 1 cm^2^ and washed again. Subsequently, the IELs were collected by incubating three times in a shaking incubator (Certomat BS-1) at 200 rpm for 15 min at 37 °C in EDTA-medium [HBSS 1x (Gibco®) supplemented with 10% heat-inactivated FCS (Lonza) and 1% 0.5 M EDTA-Na_2_ (UltraPure™, Invitrogen, the Netherlands)]. Supernatants were collected and centrifuged for 5 min at 335 × *g* at 20 °C (Allegra™ X-12R Centrifuge, Beckman Coulter, the Netherlands). Pellets were then resuspended in PBS at a concentration of 10 mL per gram tissue and an aliquot of 100 µL was used for bacteriological analysis. PBS was added to the remaining suspension up to 20 mL and IELs were isolated using Ficoll-Paque Plus (GE Healthcare, the Netherlands) density gradient centrifugation for 12 min at 673 × *g* at 20 °C, washed in PBS by centrifugation for 5 min at 393 × *g* at 4 °C and resuspended at 4.0 × 10^6^ cells/mL in complete medium (IMDM 2 mM glutamax I supplemented with 8% heat-inactivated FCS (Lonza), 2% heat-inactivated chicken serum, 100 U/mL penicillin and 100 µg/mL streptomycin; Gibco®). Spleens were homogenized using a 70 µm cell strainer (Beckton Dickinson (BD) Biosciences, NJ, USA) and the single-cell suspension was diluted in PBS at a concentration of 10 mL per gram tissue. An aliquot of 100 µL was used again for bacteriological analysis. Next, leukocytes were isolated by Ficoll-Paque Plus density gradient centrifugation (20 min, 1126 × *g*, 20 °C), washed in PBS and resuspended at 4.0 × 10^6^ cells/mL in complete medium as described for ileum.

Whole blood was allowed to coagulate by leaving it undisturbed for 1 h at room temperature (RT), centrifuged for 10 min at 2095 × *g* at 15 °C and subsequently, serum was collected and stored at −20 °C until further use.

### Quantitative bacteriology of ileum and spleen

At − 4, 0, 1, 3, 7, 14 and 21 dpi, the numbers of *Salmonella* colonies in ileum and spleen were determined by plating 100 μL of the cell suspensions of either the ileum segments or homogenized spleens with a spatula on RAPID’ *Salmonella* Medium plates (Bio-Rad, the Netherlands). Plates were incubated overnight at 37 °C and subsequently, purple colonies were quantified and SE was expressed as CFU per gram tissue. The limit of detection (LOD) was 100 CFU per gram tissue.

### Phenotypic characterization of lymphocytes by flow cytometry

Presence and activation of NK and T cell subsets were determined among IELs and splenocytes at 0, 1, 3, 7, 14 and 21 dpi as described previously [12, 42]. Lymphocyte populations (1 × 10^6^) were stained with a panel of antibodies specific for surface markers known to be expressed on NK cells, as well as with anti-CD3 to exclude T cells from the analyses. In addition, cells were stained with a panel of antibodies specific for surface markers that distinguishes γδ T cell, CD4^+^ and CD8^+^ T cell subsets (Table 1). Staining with primary and secondary antibodies was performed in 50 µL PBS (Lonza) containing 0.5% bovine serum albumin and 0.1% sodium azide (PBA). Cells were incubated for 20 min at 4 °C in the dark, washed twice by centrifugation for 5 min at 393 × *g* at 4 °C in PBA, after primary staining, and in PBS after secondary staining. Subsequently, to be able to exclude dead cells from analysis, lymphocytes were stained in 100 µL PBS with a viability dye (Zombie Aqua™ Fixable Viability Kit, Biolegend, the Netherlands) for 15 min at RT in the dark, washed twice in PBA and resuspended in 200 µL PBA. Of each sample, either 150 µL or a maximum of 1 × 10^6^ viable cells were analyzed using a CytoFLEX LX Flow Cytometer (Beckman Coulter), and data was analyzed with FlowJo software (FlowJo LCC, BD Biosciences). The gating strategies used to analyze NK cells, γδ T cells and cytotoxic CD8^+^ T cells are depicted in Additional file 1.

**Table 1:** **Flow cytometry staining reagents**

Cell population	Primary antibody (mouse-anti-chicken)	Clone/isotype	Secondary antibody
NK cells	CD45-FITC^1^	LT40/IgM	–
	CD3-APC^1^	CT3/IgG1	–
	IL-2Rα-UNL^2^	28-4/IgG3	Goat-anti-mouse-IgG3-PE^1^
	20E5-BIOT^2^	IgG1	Streptavidin (SA)-PercP^6^
T cells	CD3-PE^1^	CT3/IgG1	–
	CD4-APC^1^	CT4/IgG1	–
	TCRγδ-FITC^1^	TCR-1/IgG1	–
	CD8α-UNL^1^	EP72/IgG2b	Goat-anti-mouse-IgG2b-APC/Cy7^1^
	CD8β-BIOT^1^	EP42/IgG2a	SA-PercP^6^
APCs	CD41/61-FITC^4^	11C3/IgG1	–
	Bu-1-AF647^1^	AV20/IgG1	–
	CD3-FITC^1^	CT3/IgG1	–
	CD4-PE/Cy7^1^	CT4/IgG1	–
	MRC1LB-PE^1^	KUL01/IgG1	–
	CD11-biotin^2^	5C7/IgG1	SA-Brilliant Violet (BV) 605^7^
	MHC-II-UNL^1^	Cia/IgM	Rat-anti-mouse-IgM-BV421 (RMM-1)^7^
	*CHIR-AB1-UNL^2^	8D12/IgG2a	Rat-anti-mouse-IgG2a-PerCP/Cy5.5 (RMG2a-62)^7^
	*CD40-UNL^5^	AV79/IgG2a	Rat-anti-mouse-IgG2a-PerCP/Cy5.5 (RMG2a-62)^7^
	*CD80-UNL^5^	IAH:F864:DC7/IgG2a	Rat-anti-mouse-IgG2a-PerCP/Cy5.5 (RMG2a-62)^7^
Assay			
CD107	CD107a-APC^3^	LEP-100 I 5G10/IgG1	–
	CD41/61-FITC^4^	11C3/IgG1	–
	CD3-PE^1^	CT3/IgG1	–
	CD8α-UNL^1^	EP72/IgG2b	Goat-anti-mouse-IgG2b-Alexa Fluor (AF) 790^8^
	28–4-UNL^2^	IgG3	Goat-anti-mouse-IgG3-APC/Cy7^1^
	20E5-BIOT^2^	IgG1	SA-PercP^6^
IFNγ	CD3-PE^1^	CT3/IgG1	–
	TCRγδ-FITC^1^	TCR-1/IgG1	–
	CD8α-UNL^1^	EP72/IgG2b	Goat-anti-mouse-IgG2b-AF790^8^
	28-4-UNL^2^	IgG3	Goat-anti-mouse-IgG3-APC/Cy7^1^
	20E5-BIOT^2^	IgG1	SA-PercP^6^
	IFNγ-APC^3^	MAb80/IgG1	–
T cell proliferation	CD3-FITC^1^	CT3/IgG1	–
	CD4-PE/Cy7^1^	CT4/IgG1	–
	CD8α-APC^1^	CT8/IgG1	–
	IL-2Rα-UNL^2^	28-4/IgG3	Goat-anti-mouse-IgG3-PE^1^

Three APC antibody panels were prepared each containing surface markers plus one out of three antibodies indicated (*).

Manufacturer: ^1^Southern Biotech, AL, USA.

^2^Purified antibody from hybridoma supernatant [44], kindly provided by Göbel, T.W., Ludwig Maximilian University, Germany.

^3^Developmental Studies Hybridoma Bank (DSHB), University of Iowa, IA, USA.

^4^Serotec, United Kingdom.

^5^Bio-Rad.

^6^BD Biosciences.

^7^Biolegend.

^8^Jackson ImmunoResearch Laboratories, PA, USA.

### CD107 assay

Activation of NK cells and cytotoxic CD8^+^ T cells was determined using the CD107 assay, which measures the increased surface expression of CD107a that results from degranulation, the release of cytotoxic granules [44]. Briefly, lymphocytes isolated from the IEL population and spleen were resuspended in complete medium, and 1 × 10^6^ lymphocytes in 0.5 mL were incubated in the presence of 1 µL/mL GolgiStop (BD Biosciences) and 0.5 µL/mL mouse-anti-chicken-CD107a-APC for 4 h at 37 °C, 5% CO_2_. After incubation, lymphocytes were washed in PBA and stained as described in “Phenotypic characterization of lymphocytes by flow cytometry” section with monoclonal antibodies for NK and T cells, and anti-CD41/61 to exclude thrombocytes from analyses, as mentioned in the CD107 panel (Table 1). Cells were washed in PBS, stained for viability and analyzed by flow cytometry.

### IFNγ assay

Expression of intracellular IFNγ was determined in (subsets of) NK cells, γδ T cells, CD4^+^ and CD8^+^ T cells, using the assay adapted from Ariaans et al. [45]. Lymphocytes isolated from the IEL population and spleen were resuspended in complete medium, and 1 × 10^6^ lymphocytes in 0.5 mL were incubated in the presence of 1 µL/mL Brefeldin A (Sigma Aldrich) for 4 h at 41 °C, 5% CO_2_. After incubation, lymphocytes were washed in PBA and stained as described in “Phenotypic characterization of lymphocytes by flow cytometry” section with surface markers as mentioned in the IFNγ panel (Table 1). Cells were washed in PBS, stained for viability and washed again in PBA. Then, lymphocytes were permeabilized differently as described by Ariaans et al. [45]. Lymphocytes were incubated in 200 µL of a mixture of BD FACS™ Permeabilizing Solution 2 and BD FACS™ Lysing Solution prepared according to manufacturer’s instructions (BD Biosciences) for 8 min at RT, immediately followed by centrifugation for 2 min at 393×*g* at 4 °C. Cells were washed twice in PBA, stained intracellularly with anti-IFNγ-APC in 50 µL PBA for 20 min at 4 °C in the dark, washed in PBA and finally analyzed by flow cytometry.

### Phenotypic characterization of APCs by flow cytometry

Splenocytes isolated at 0, 1, 3 and 7 dpi from infected and uninfected chickens were transferred to a 96 wells V-bottom plate and stained with antibodies of the APC panel to distinguish APC subsets (Table 1). Staining with primary and secondary antibodies (1 × 10^6^) was performed in 50 µL PBA, incubated for 20 min at 4 °C in the dark and washed twice by centrifugation for 3 min at 393 × *g* at 4 °C in PBA. Finally, the cells were stained in 50 µL PBS with ViaKrome 808 viability dye (Beckman Coulter) for 20 min at 4 °C. Cells were washed in PBA and analyzed by flow cytometry as described in “Phenotypic characterization of lymphocytes by flow cytometry” section, using 180 µL.

Based on the APC subset staining, a t-distributed Stochastic Neighbor Embedding (t-SNE) analysis was performed using FlowJo software to identify cell subsets using an unbiased approach. From each sample of splenocytes at 7 dpi of infected (*n* = 5) and uninfected (*n* = 5) chickens, 10 000 cells were taken and concatenated into one FCS file that represented all individual chickens. The t-SNE was performed based on expression levels of CD3, CD41/61, MRC1LB, CD4, Bu-1, CD11, MHC-II and CHIR-AB1 using published automated optimized parameters [46]. Based on the t-SNE, three APC subsets were identified based on selection of MRC1LB and CD11 positive cells, which were negative for CD3, CD4 and CD41/61. Activation status of the subsets was subsequently evaluated using the expression percentages of immunoglobulin Y receptor CHIR-AB1, co-stimulatory molecules CD40 and CD80, and the geometric mean fluorescent intensity (gMFI) of MHC-II. CHIR-AB1 was included as an activation marker since surface expression of this marker has been described to be induced on macrophages upon stimulation with LPS or IFN-γ, which was recently confirmed by van den Biggelaar et al. [47, 48].

### Fluorescence-activated cell sorting of NK cell and APC subsets

Based on marker expression, two NK cell subsets and three APC subsets were separated by fluorescence-activated cell sorting (FACS) to gain more insight into their functional identity. To distinguish NK cell subsets, splenocytes were stained with mouse-anti-chicken-CD3-APC, -Bu-1-FITC, -28-4 and -20E5-biotin. For secondary antibody staining goat-anti-mouse-IgG3-PE and SA-BV421 were used. To identify APC subsets, splenocytes were stained with mouse-anti-chicken-MRC1LB-PE and -CD11-biotin. Secondary staining with SA-BV605 was used to fluorescently label CD11-biotin. To assess viability, the cells were stained with the Zombie Aqua™ Fixable Viability Kit. Primary and secondary antibody staining of cells used the same conditions as described in “Phenotypic characterization of lymphocytes by flow cytometry” section. Finally, the cells were resuspended in PBA (NK cells) or PBA with 2 mM EDTA-Na_2_ (APC subsets), and isolated by FACS using the BD influx™ Cell Sorter and 405-, 488-, 638-, 561- and 640-nm lasers. The cells were gated for viability and subsequently sorted into CD3^−^ Bu-1^−^ 28–4^+^ (IL-2Rα^+^ NK), CD3^−^ Bu-1^−^ 20E5^+^ (20E5^+^ NK), respectively CD11^+^ MRC1LB^+^ (APC subset 1), CD11^+^ MRC1LB^−^ FSC^low^ (APC subset 2a) and CD11^+^ MRC1LB^−^ FSC^high^ (APC subset 2b). The sorted NK cell subsets were collected in 350 µL RLT buffer (Qiagen, the Netherlands) with 1% 2-mercaptoethanol (Sigma Aldrich). The sorted APC subsets, and the original (unsorted) cell population as a control, were centrifuged at 393 × *g* for 5 min and then lysed in 600 µL RLT buffer with 1% 2-mercaptoethanol. Cell lysates of sorted cell subsets and the control cell population were then stored at −20 °C until RNA isolation and qPCR analysis.

### Gene expression of separated subsets of NK cells and APCs

Target genes (Table 2) were selected based on literature to define functional differences between subsets of NK cells respectively APCs. RNA was isolated from lysates of sorted NK and APC subsets, and control cells, using the RNeasy Mini Kit (Qiagen) according to the manufacturer’s instructions, including a DNase treatment using the RNase-Free DNase Set (Qiagen). Next, cDNA was prepared using the reverse transcriptase from the iScript cDNA Synthesis Kit (Bio-Rad) according to the manufacturer's instructions. RT-qPCRs were performed with primers and either FAM-TAMRA-labeled TaqMan probes combined with TaqMan Universal PCR Master Mix or SYBR Green Master Mix without probes (all from Thermo Fisher Scientific), as indicated in Table 2. Primers were used at 400 nM (SYBR-Green) or 600 nM (Taqman) and probes at 100 nM. RT-qPCRs were performed with a CFX Connect and analyzed with CFX Maestro software (both from Bio-Rad). All RT-qPCRs were evaluated for proper amplification efficiency (95–105%) using serial dilutions of reference cDNA either from splenocytes that were stimulated with Concanavalin A for 24 h or from HD11 cells that were stimulated with LPS for 3 h. RT-qPCRs were performed in triplicate for every sample. For the NK cell subsets, mRNA levels are expressed as 40-Ct and the cycle threshold value (Ct) was corrected for variations in RNA preparation and sampling using the *GAPDH* Ct values, as described elsewhere [49]. Higher gene expression of *NFIL3* and *IL-7α* is indicative of the cytokine-producing NK cell subset in humans [50–54], whereas higher expression of *PRF1* is indicative of the cytotoxic NK cell subset in humans and chickens [52, 55]. Gene expression levels of the APC subsets are shown relative to those of unsorted splenocytes. Furthermore, Ct gene expression values were normalized to housekeeping genes *28S* and *GAPDH*. Changes in gene expression after sorting was expressed as 2^−ΔΔCt^, according to the Livak method [56]. Enrichment of cells expressing *CD14*, *TLR4*, *MERTK* and *MAFB* after sorting of cells was considered indicative for a monocyte/macrophage phenotype, whereas enrichment of cells expressing *ZBTB46*, *XCR1* and *FLT3* after sorting was considered indicative for a DC phenotype, in accordance with previous studies [57, 58].

**Table 2:** **Primers and TaqMan probe sequences used for RT-qPCR**

Cell type	Genes	NCBI Reference	TaqMan/SYBR-Green	Type	Sequence (5ʹ-3ʹ)
NK cells	*NFIL3*	XM_017014743.1	SYBR-Green	Forward	TGAATGCCATCAGTTGAGC
				Reverse	GAGAGGCGGAGAATGTGAGT
	*IL-7Rα*	NM_001080106.1	SYBR-Green	Forward	ATTCTGGGAAAGCAGGATCAAG
				Reverse	CTTACACAGTCGCTCCAGAGTTATTT
	*PRF1*	XM_004945690.3	SYBR-Green	Forward	ACCCGCACCAAAAGATGAAG
				Reverse	TAATTCGCACACCCCTAAACG
	*GAPDH*	NM_204305.1	SYBR-Green	Forward	GTGGTGCTAAGCGTGTTATC
				Reverse	GCATGGACAGTGGTCATAAG
APCs	*CD14*	NM_001139478.1	TaqMan	Forward	GGACGACTCCACCATTGACAT
				Reverse	GGAGGACCTCAGGAACCAGAA
				Probe	AATGATCTTCCTGATTTGCAGACTGCCAA
	*TLR4*	NM_001030693.1	SYBR-Green	Forward	GTCCCTGCTGGCAGGAT
				Reverse	TGTCCTGTGCATCTGAAAGCT
	*MERTK*	NM_204988.1	SYBR-Green	Forward	TGTGGAAGGATGGCAGGGAG
				Reverse	GCACGGATGCTGAATGTAGAGG
	*MAFB*	NM_001030852.1	SYBR-Green	Forward	AGGACCGGTTCTCGGATGAC
				Reverse	CCTCGGAGGTGCCTGTTG
	*INOS*	NM_204961.1	SYBR-Green	Forward	TGGGTGGAAGCCGAAATA
				Reverse	GTACCAGCCGTTGAAAGGAC
	*ZBTB46*	XM_015296613.2	SYBR-Green	Forward	CTGGACCTGTGGAAGAGGAAAC
				Reverse	CGGTAGTGGGAGGCAATCTC
	*XCR1*	NM_001024644.2	SYBR-Green	Forward	CCTTCGGGTGGATTTTTGGT
				Reverse	CGCTGTAGTAGCCAATGGAGAA
	*FLT3*	NM_004119.3	SYBR-Green	Forward	CATTCGGACCCAGTACATGTTTAC
				Reverse	TGAGCCGTAGAAGAGCAGGTATAA
	*GAPDH*	NM_204305.1	SYBR-Green	Forward	GTGGTGCTAAGCGTGTTATC
				Reverse	GCATGGACAGTGGTCATAAG
	*28S*	XR_00378040.1	TaqMan	Forward	GGCGAAGCCAGAGGAAACT
				Reverse	GACGACCGATTTGCACGTC
				Probe	AGGACCGCTACGGACCTCCACCA

All sequences have been described previously except for *NFIL3* and *PRF1* [57, 58, 79].

### T cell proliferation assay

Splenocytes isolated at 21 dpi from uninfected and SE-infected chickens were labelled with CellTrace Violet (CTV, Invitrogen) to measure proliferation by flow cytometry. The cells were resuspended at 5 × 10^6^ cells/mL in PBS with 5 µM CTV and incubated for 20 min at RT, while the cell suspension was vortexed every 5 min. Next, the labeling was quenched by the addition of 5 mL complete medium for every mL of CTV staining solution and incubated for 5 min at RT. Cells were centrifuged for 5 min at 335 × *g* at 20 °C and resuspended at 2.5 × 10^6^ cells/mL in X-VIVO 15 cell culture medium (Lonza) with 50 U/mL penicillin–streptomycin, 50 µM 2-mercaptoethanol (Sigma Aldrich) and 50 µg/mL gentamycin (Gibco™). Aliquots of 200 µL cell suspension containing 500 000 splenocytes were added to the wells of a 96 wells round-bottom cell culture plate. Fixed SE was added to the splenocytes at 10^4^, 10^5^ or 10^6^ CFU/well. As a positive control, splenocytes were stimulated with 1 µg/mL mouse-anti-chicken-CD3, 1 µg/mL-CD28 and 1:50 diluted conditioned supernatant from COS-7 cells transfected with a pcDNA1 vector (Invitrogen) encoding for recombinant chicken IL-2 (a kind gift from prof. Pete Kaiser and Lisa Rothwell), in accordance with a previous publication [59]. Cells were incubated for 4 days at 41 °C and 5% CO_2_. After incubation, cells were transferred to a 96 wells V-bottom plate and stained in PBS with ViaKrome 808 viability dye (Beckman Coulter). Next, cells were stained with antibodies of the T cell proliferation panel (Table 1). Primary and secondary staining of cells were conducted in 30 µL PBA and incubated for 20 min at 4 °C in the dark. Stained cells were washed twice by centrifugation for 3 min at 393 × *g* at 4 °C in PBA and resuspended in 100 µL followed by flow cytometry analysis as described in “Phenotypic characterization of lymphocytes by flow cytometry” section, using 80 µL.

### SE-specific antibody titers in serum

To detect titers of SE-specific antibodies in the sera collected at 0, 7, 14 and 21 dpi, the commercially available *Salmonella* Enteritidis Antibody Test (IDEXX SE Ab X2 Test) was used according to manufacturer’s instructions (IDEXX Europe, the Netherlands). Positive and negative controls were included in the kit, and serum samples were analyzed in duplicate. Endpoint titers were calculated with the following formula:

$$10^{{(1.5 \, \times \log 10(({\text{sample}}\;\upmu - {\text{negative}}\;{\text{control}}\;\upmu )/({\text{positive}}\;\upmu - {\text{negative}}\;{\text{control}}\;\upmu )) + 3.47)}} .$$

### Statistical analysis

First, the data were tested for fitting a normal distribution using the Shapiro–Wilk test. Differences in numbers of IELs or leukocytes, NK cell and T cell subsets and percentages of CD107 and IFNγ expression in the IELs and spleen between the uninfected and SE-infected groups as well as within each group in the course of time were analyzed using one-way ANOVA tests. Differences in SE CFUs per gram ileum and spleen as well as SE-specific antibody titers in serum were analyzed using Kruskal–Wallis tests accompanied by Dunn’s multiple comparisons tests. Differences in numbers and percentages of the splenic APC subsets 1 and 2a between the uninfected and SE-infected groups were analyzed using one-way ANOVA tests, while subset 2b was analyzed using the Kruskal–Wallis test as the data was not normally distributed. All statistical analyses were performed using GraphPad Prism 9 software (GraphPad Software, CA, USA). A *p*-value of < 0.05 was considered statistically significant and a value of 0.05 < *p* < 0.1 is referred to as a trend, in case the *p*-value did not belong to one of these categories it is referred to as a numerical difference.

## Results

### Highest presence of SE in ileum and spleen at 7 dpi while intestinal infiltration of IELs was observed at 1 dpi

**Figure 1:**
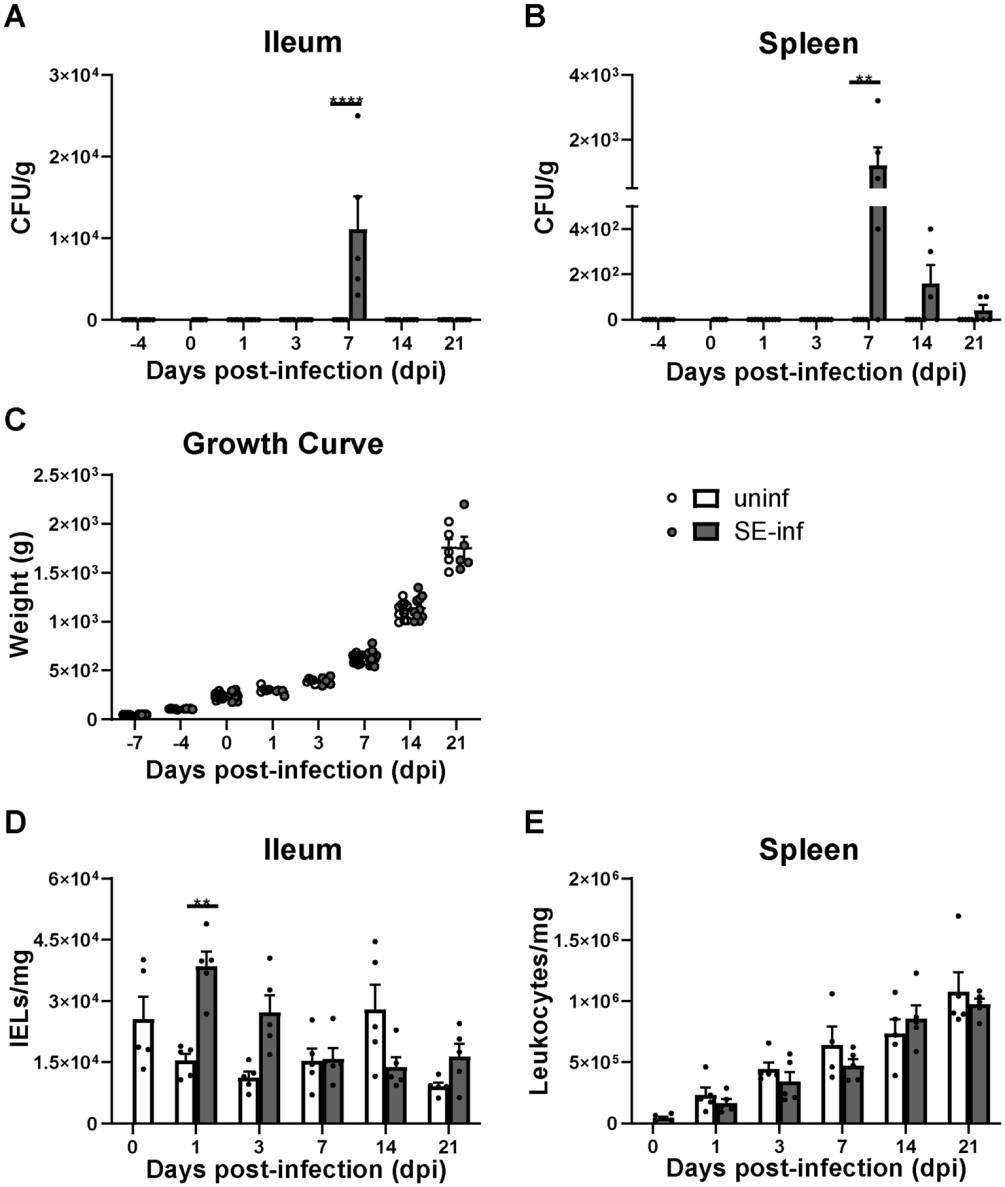
**The bacterial load in course of time after SE infection and its effect on growth and numbers of IELs and splenic leukocytes.A***Salmonella enterica* serotype Enteritidis (CFU/g) in the ileum and **B** spleen of uninfected (uninf) and SE-infected chickens (SE-inf). The LOD was 100 CFU per gram tissue. **C** Bodyweights (g) of uninfected and SE-infected chickens in the course of time. **D** Numbers (cells/mg) of IELs per mg ileum and **E** leukocytes per mg spleen of uninfected and SE-infected chickens in the course of time. Mean + SEM per treatment and time point is shown (*n* = 5) and statistical significance is indicated as ** *p* < 0.01 and **** *p* < 0.0001.

SE was not observed at − 4 and 0 dpi before SE inoculation, in both the IEL population of the ileum and the spleen of chickens of both groups (Figures 1A and B). After inoculation SE was detected in the ileum of SE-infected chickens only at 7 dpi (Figure 1A). In the spleen, SE was observed at 7, 14 and 21 dpi with the highest bacterial counts at 7 dpi, which subsequently decreased in course of time (Figure 1B). SE was not detected in the ileum and spleen of uninfected chickens at any of the time points (Figures 1A and B). One uninfected chicken showed counts of *Proteus* in the spleen at 7 dpi and was therefore excluded from further analyses. Infection with SE did not affect the weight of the chickens, as growth curves were similar between uninfected and SE-infected chickens (Figure 1C). A significant increase in numbers of IELs was found in SE-infected chickens at 1 dpi compared to uninfected chickens (Figure 1D). IELs were numerically higher at 3 dpi in SE-infected chickens and declined over time to numbers similar to those observed in uninfected chickens (Figure 1D). The numbers of splenic leukocytes were similar between uninfected and SE-infected chickens at all time points (Figure 1E).

### Enhanced activation of intraepithelial NK cells upon SE infection

**Figure 2:**
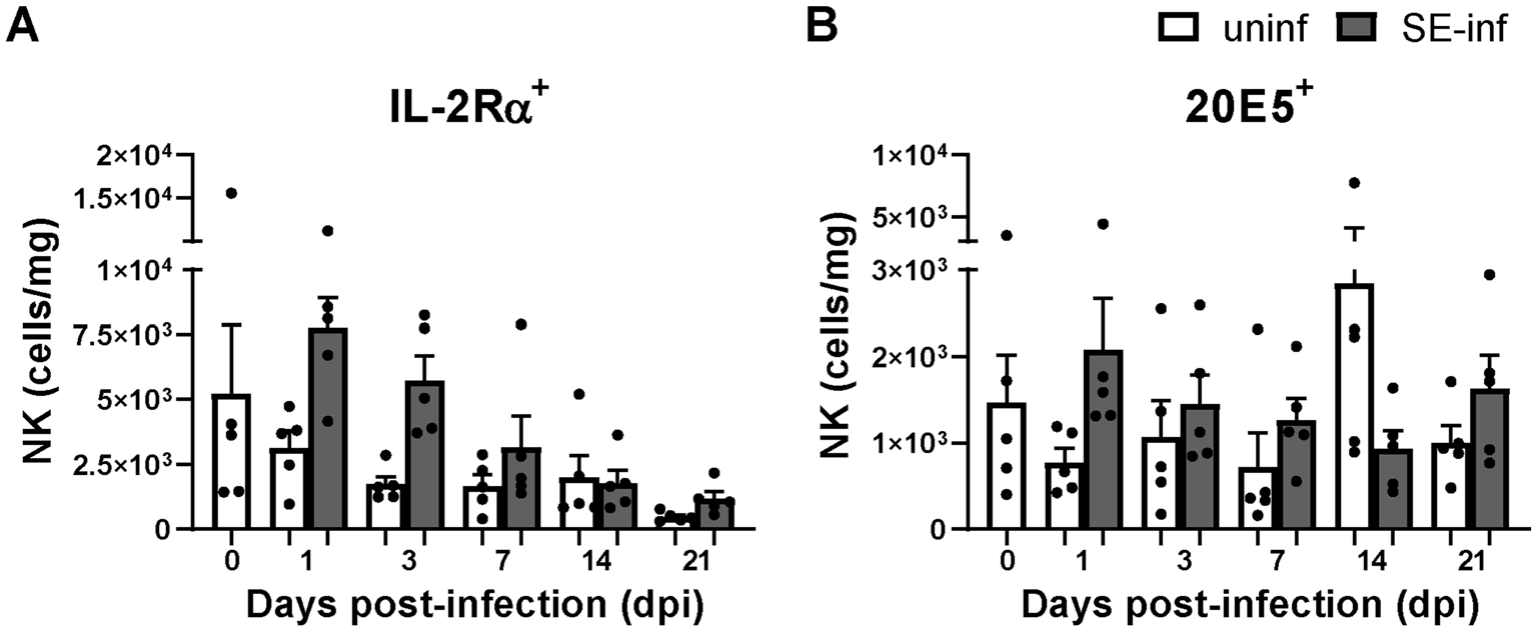
**Effect of SE infection on numbers of intraepithelial NK cells in broiler chickens.A** Numbers (cells/mg) of intraepithelial IL-2Rα^+^ and **B** 20E5^+^ NK cells per mg ileum, in uninfected (uninf) and SE-infected (SE-inf) chickens. Mean + SEM per treatment and time point is shown (*n* = 5).

Numbers of intraepithelial and splenic NK cell subsets were determined to investigate differences between uninfected and SE-infected chickens at several time points post-infection. NK cell subsets were distinguished by membrane expression of IL-2Rα or 20E5 (Additional file 1). Although no significant differences were observed in numbers of intraepithelial NK cell subsets, IL-2Rα^+^ and 20E5^+^ NK cells were numerically higher in SE-infected chickens at 1, 3 and 7 dpi compared to uninfected chickens (Figures 2A and B). Furthermore, intraepithelial 20E5^+^ NK cells were numerically lower at 14 dpi and higher at 21 dpi in SE-infected compared to uninfected chickens (Figure 2B). In the control group, no significant differences were observed in the numbers of intraepithelial NK cells in course of time. In the spleen, numbers of IL-2Rα^+^ and 20E5^+^ NK cells were similar between uninfected and SE-infected chickens and both increased in course of time (Additional files 2A and B). To obtain more insight in functional differences between IL-2Rα^+^ and 20E5^+^ NK cells, mRNA levels of genes deemed to be relevant were determined in the spleen. The IL-2Rα^+^ subset showed numerical higher mRNA levels of the NK cell lineage marker NFIL3 and IL-7Rα^+^ as compared to the 20E5^+^ subset, whereas the 20E5^+^ subset showed a numerical higher mRNA level of perforin as compared to the IL-2Rα^+^ subset (PRF1, Additional file 2C).

**Figure 3:**
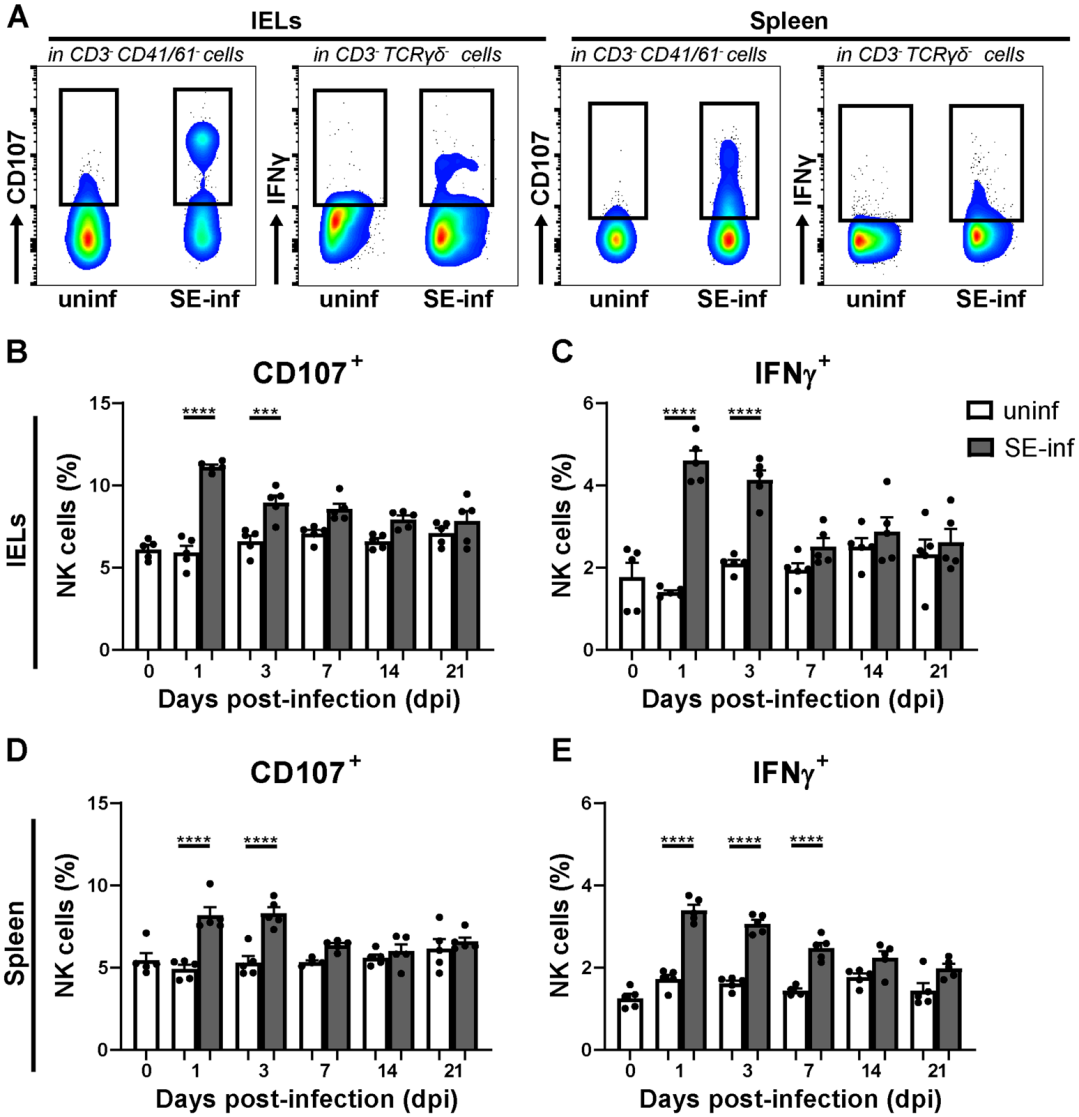
**NK cell activation in the IEL population and spleen of broiler chickens upon SE infection.A** Gating strategy for NK cells expressing surface CD107 and intracellular IFNγ in the IEL population (first and second panels) and spleen (third and fourth panels). **B** Percentages of intraepithelial NK cells expressing CD107 and **C** IFNγ in uninfected (uninf) and SE-infected (SE-inf) chickens in the course of time. **D** Percentages of splenic NK cells expressing CD107 and **E** IFNγ in uninfected and SE-infected chickens. Mean + SEM per treatment and time point is shown (*n* = 5), for uninfected chickens at 7 dpi in spleen *n* = 4. Statistical significance is indicated as *** *p* < 0.001 and **** *p* < 0.0001.

To determine possible changes in NK cell activation upon SE infection, CD107 surface expression and intracellular IFNγ were analyzed in intraepithelial and splenic NK cells (Figure 3A). Intraepithelial NK cells showed a significant increase in surface expression of CD107 and IFNγ production in SE-infected chickens at 1 dpi and 3 dpi compared to uninfected chickens (Figures 3B and C). In the spleen, a significant increase in surface expression of CD107 was observed at 1 dpi and 3 dpi, and a significant increase in IFNγ production was observed at 1 dpi, 3 dpi and 7 dpi in SE-infected compared to uninfected chickens (Figures 3D and E).

### Increased presence of APCs in the spleens of SE-infected chickens

**Figure 4:**
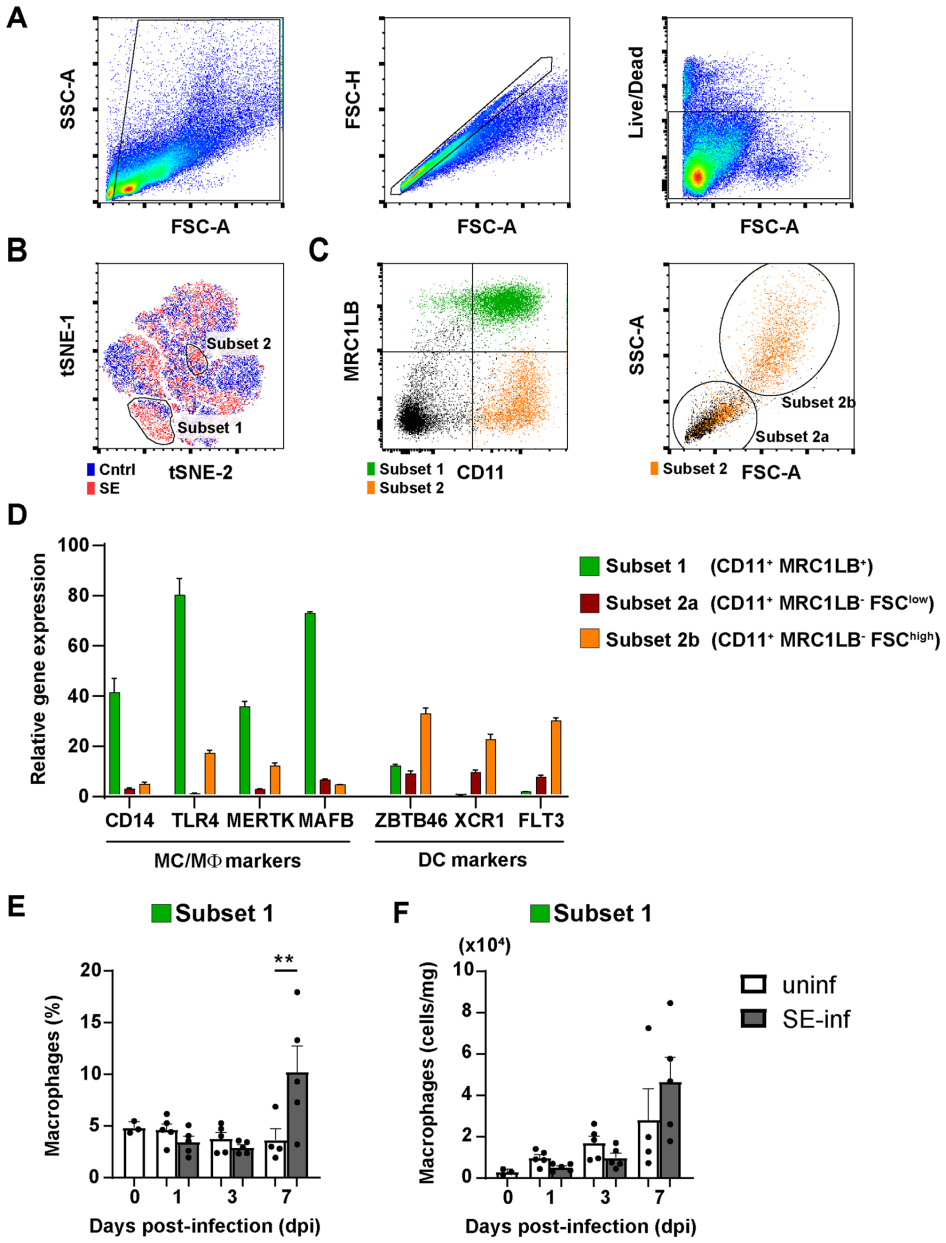
**Phenotypic characterization of splenic APCs upon SE infection.A** Splenocytes were gated for size, excluding debris (FSC-A vs SSC-A), singlets (FSC-A vs FSC-H) and viability (Live/Dead marker-negative) consecutively. **B** A t-SNE analysis was performed on spleen samples of 7 dpi uninfected (uninf, blue) and SE-infected (SE-inf, red) chickens combined. Based on the t-SNE analysis, two population (subset 1 and subset 2) were found enriched among the splenocytes of SE-infected chickens. **C** The populations were evaluated for expression of MRC1LB versus CD11. Subset 2 was evaluated for its FSC-A vs SSC-A scatter pattern and further subdivided into subset 2a and subset 2b. **D** Subset 1 (CD11^+^ MRC1LB^+^), subset 2a (CD11^+^ MRC1LB^−^ FSC^low^) and subset 2b (CD11^+^ MRC1LB^−^ FSC^high^) were sorted by FACS to assess gene expression of immune markers by RT-qPCR relative to the total splenocyte population. **E** The presence (%) and **F** numbers (cells/mg spleen) of macrophages in uninfected and SE-infected chickens were assessed over time. Mean + SEM per treatment and time point is shown (*n* = 5), for uninfected chickens at 0 dpi *n* = 3 and at 7 dpi *n* = 4. Statistical significance is indicated as ** *p* < 0.01.

To investigate whether infection with SE affects the composition of the APC population, amongst splenocytes, these were stained for APC surface markers and analyzed by flow cytometry. A t-SNE analysis was used to determine the differences in APCs between uninfected and SE-infected chickens (Figures 4A–C and Additional file 3A). This analysis clustered cells that have high similarity and separated cells that are unrelated based on the APC surface markers that were used, leading to an unbiased visualization of all cell populations. Two populations were found overrepresented in the spleen of SE-infected chickens (Figure 4B). By gating for subset 1 and 2 and assessing their expression of APC markers, subset 1 was identified as CD11^+^ MRC1LB^+^ and subset 2 as CD11^+^ MRC1LB^−^ (Figure 4C, first panel). In addition, subset 2 could be further divided into two subsets, distinguished by FSC-A and SSC-A characteristics, that were further analyzed separately (Figure 4C, second panel).

The APC subsets were sorted (Additional files 3B and C), and qPCR was performed to compare the expression levels of macrophage- and DC-specific genes between sorted cells and the unsorted total APC population. High expression levels of the monocyte/macrophage genes CD14, TLR4, MERTK and MAFB (Figure 4D) observed on the CD11^+^ MRC1LB^+^ cells, indicates that this subset 1 includes macrophages (hereafter referred to as macrophages). The CD11^+^ MRC1LB^−^ FSC^high^ subset 2b includes DCs as reflected by high expression of the DC genes ZBTB46, XCR1 and FLT3 (hereafter referred to as FSC^high^ DCs) (Figure 4D). The increase in expression of either macrophage- or DC-specific genes was less clear in the CD11^+^ MRC1LB^−^ FSC^low^ subset 2a, however, DC-specific genes were most abundantly expressed (hereafter referred to as FSC^low^ DCs) (Figure 4D).

Next, the percentages (Figure 4E and Additional file 4A and B) and numbers (Figure 4F and Additional file 4C and D) of the three APC subsets were followed over time in the spleens of uninfected and SE-infected chickens. Due to limited cell numbers, the analysis of APCs could not be performed for two chickens at 0 dpi. At 7 dpi, the percentage of macrophages was significantly increased in SE-infected compared to uninfected chickens (Figure 4E). The FSC^low^ (Additional file 4A and C) and FSC^high^ (Additional file 4B and D) DCs were similar in SE-infected compared to uninfected chickens at all time points, although a slight increase in both the percentages and numbers of FSC^high^ DCs was observed at 7 dpi (Additional file 4B and D).

### APCs become activated in spleens of SE-infected chickens

**Figure 5:**
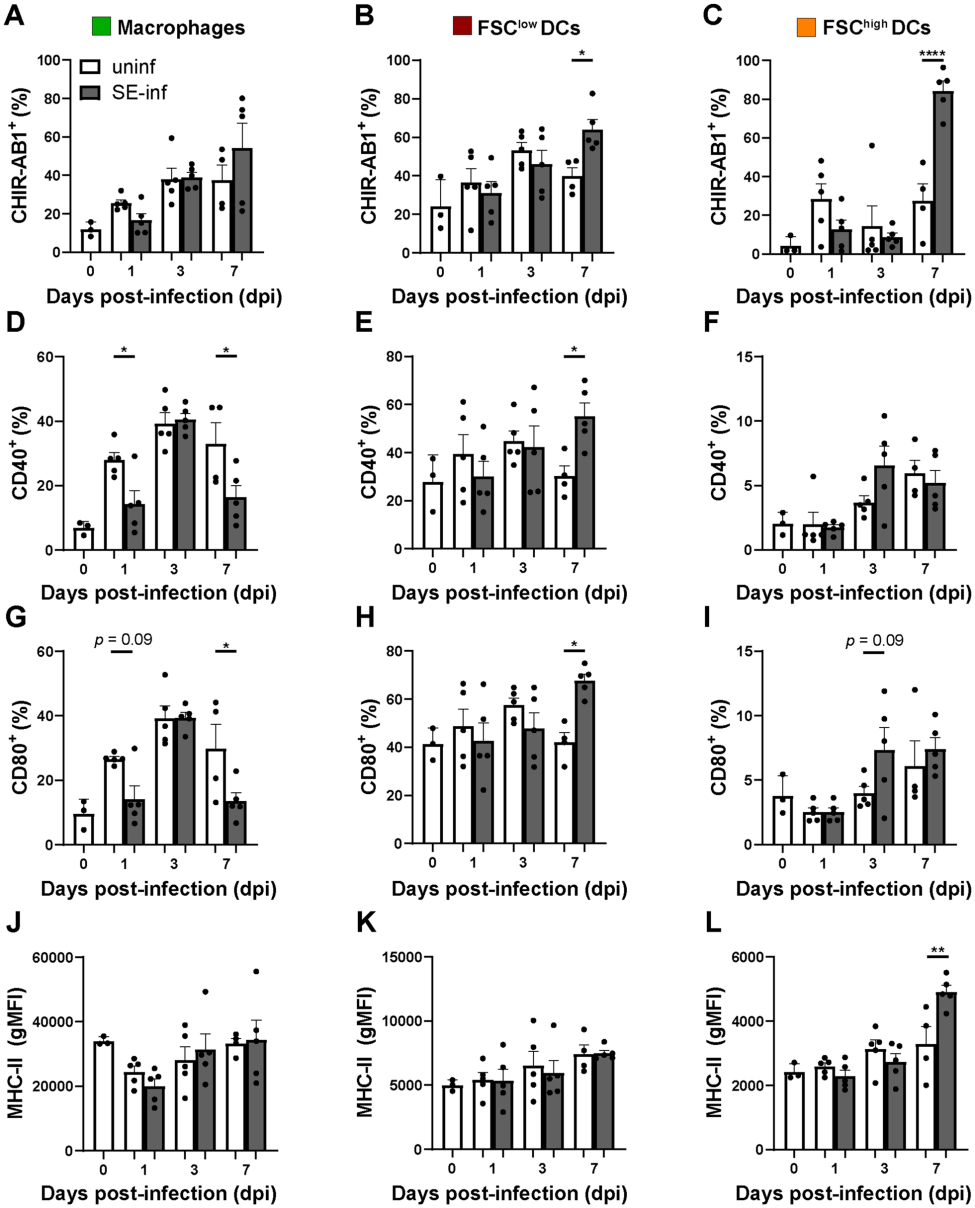
**Activation marker expression by splenic APC subsets upon SE infection.A** Macrophages, **B** FSC^low^ DCs and **C** FSC^high^ DCs were assessed over time for CHIR-AB1, **D-F** CD40, **G-I** CD80 and **J-L** MHC-II expression in uninfected (uninf) and SE-infected (SE-inf) chickens. For CHIR-AB1, CD40 and CD80, the percentage of cells in each APC subset expressing the respective markers is shown, and for MHC-II the geometric mean fluorescent intensity (gMFI) of each subset, in accordance with the gating strategy depicted in Additional file 5. Mean + SEM per treatment and time point is shown (*n* = 5), for uninfected chickens at 0 dpi *n* = 3 and at 7 dpi *n* = 4. Statistical significance is indicated as * *p* < 0.05, ** *p* < 0.01, **** *p* < 0.0001.

To assess the activation status of the three APC subsets in response to SE infection, expression levels of immunoglobulin Y receptor CHIR-AB1, co-stimulatory molecules CD40 and CD80, and MHC-II were evaluated by flow cytometry (Additional file 5). The macrophages of SE-infected chickens showed significantly decreased expression of the activation markers CD40 (1 and 7 dpi) and CD80 (7 dpi), whereas expression of CHIR-AB1 and MHC-II was similar compared to uninfected chickens (Figure 5A, D, G, J). Before infection, FSC^low^ DCs of uninfected chickens showed a higher expression of MHC-II (Figure 5K, L) and more cells that were positive for the costimulatory molecules CD40 (Figure 5E, F) and CD80 (Figure 5H, I) compared to FSC^high^ DCs. At 7 dpi, FSC^low^ DCs showed significantly increased expression of CHIR-AB1 (Figure 5B), CD40 (Figure 5E) and CD80 (Figure 5H) in SE-infected chickens as compared to uninfected chickens. The FSC^high^ DCs showed at 7 dpi significantly increased expression of CHIR-AB1 (Figure 5C) and MHC-II (Figure 5L) in SE-infected compared to uninfected chickens. In course of time, expression of CD40 by macrophages significantly increased at 1 dpi, 3 dpi and 7 dpi as compared to 0 dpi in the control group (Figure 5D). In addition, CD80 expression by macrophages significantly increased at 3 dpi as compared to 0 dpi in the control group (Figure 5G).

### Increased presence of intraepithelial cytotoxic T cells at 1 dpi and proliferation of SE-induced splenic T cells ex vivo at 21 dpi

**Figure 6:**
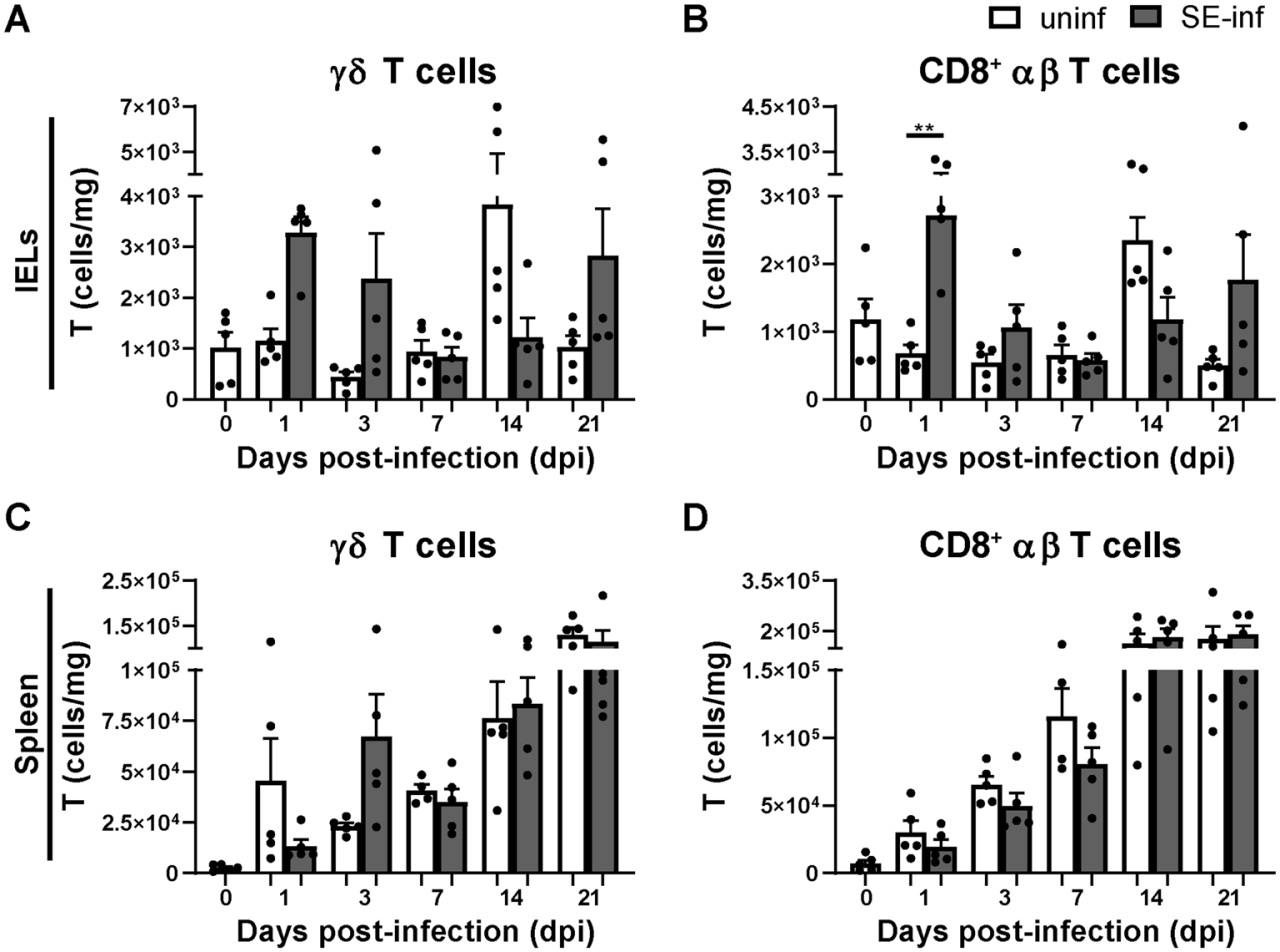
**Numbers of intraepithelial and splenic T cells in broiler chickens upon SE infection.A** Numbers (cells/mg) of intraepithelial γδ T cells and **B** CD8^+^ αβ T cells per mg ileum in uninfected (uninf) and SE-infected (SE-inf) chickens. **C** Numbers (cells/mg) of splenic γδ T cells and **D** CD8^+^ αβ T cells per mg spleen in uninfected and SE-infected chickens. Mean + SEM per treatment and time point is shown (*n* = 5), for uninfected chickens at 7 dpi in spleen *n* = 4. Statistical significance is indicated as ** *p* < 0.01.

Numbers of γδ T cells and cytotoxic (CD8^+^) αβ T cells were determined in course of time in the IEL population and spleen of uninfected and SE-infected chickens (Additional file 1). Although numbers of intraepithelial γδ T cells did not significantly differ, they were numerically higher at 1 and 3 dpi, as well as at 21 dpi in SE-infected compared to uninfected chickens (Figure 6A). A significant increase in numbers of intraepithelial cytotoxic CD8^+^ T cells was observed at 1 dpi, and at 3 dpi and 21 dpi intraepithelial cytotoxic CD8^+^ T cells were numerically higher in SE-infected compared to uninfected chickens (Figure 6B). In the spleen, γδ T cells were numerically decreased at 1 dpi but increased at 3 dpi in SE-infected compared to uninfected chickens (Figure 6C). Numbers of splenic cytotoxic CD8^+^ T cells were similar between uninfected and SE-infected chickens during the course of infection (Figure 6D). Next, γδ T cells and cytotoxic αβ T cells were analyzed for their CD8αα and CD8αβ expression (Additional file 1). Numbers of intraepithelial CD8αα^+^ γδ T cells (Additional file 6A) were significantly increased at 1 dpi and 21 dpi, and CD8αβ^+^ γδ T cells (Additional file 6B) were numerically higher at those time points in SE-infected compared to uninfected chickens. Similarly, numbers of intraepithelial cytotoxic CD8αα^+^ T cells (Additional file 6C) were significantly increased at 1 dpi and they were numerically higher at 21 dpi. The cytotoxic CD8αβ^+^ T cells (Additional file 6D) were numerically higher at those time points in SE-infected compared to uninfected chickens. In the spleen, CD8αα^+^ γδ T cell numbers (Additional file 6E) were significantly increased at 14 dpi, whereas numbers of CD8αβ^+^ γδ T cells (Additional file 6F) were similar in SE-infected versus uninfected chickens. Numbers of splenic cytotoxic CD8αα^+^ (Additional file 6G) and CD8αβ^+^ (Additional file 6H) T cells as well helper CD4^+^ T cells (Additional file 7) were similar between uninfected and SE-infected chickens during the course of infection. Finally, no significant differences were observed in T cell activation, determined by CD107 and IFNγ expression, in the IEL population and spleen between uninfected and SE-infected chickens (Additional file 8). Although expression of CD107 was numerically higher at 3 dpi by intraepithelial and splenic CD8^+^ T cells (comprising both γδ and αβ TCRs, Additional file 8A and B respectively), as well as expression of IFNγ by splenic CD4^+^ T cells (Additional file 8D) in SE-infected compared to uninfected chickens. Expression of IFNγ by T cell subsets in the IEL population could not be determined due to too low cell numbers.

**Figure 7:**
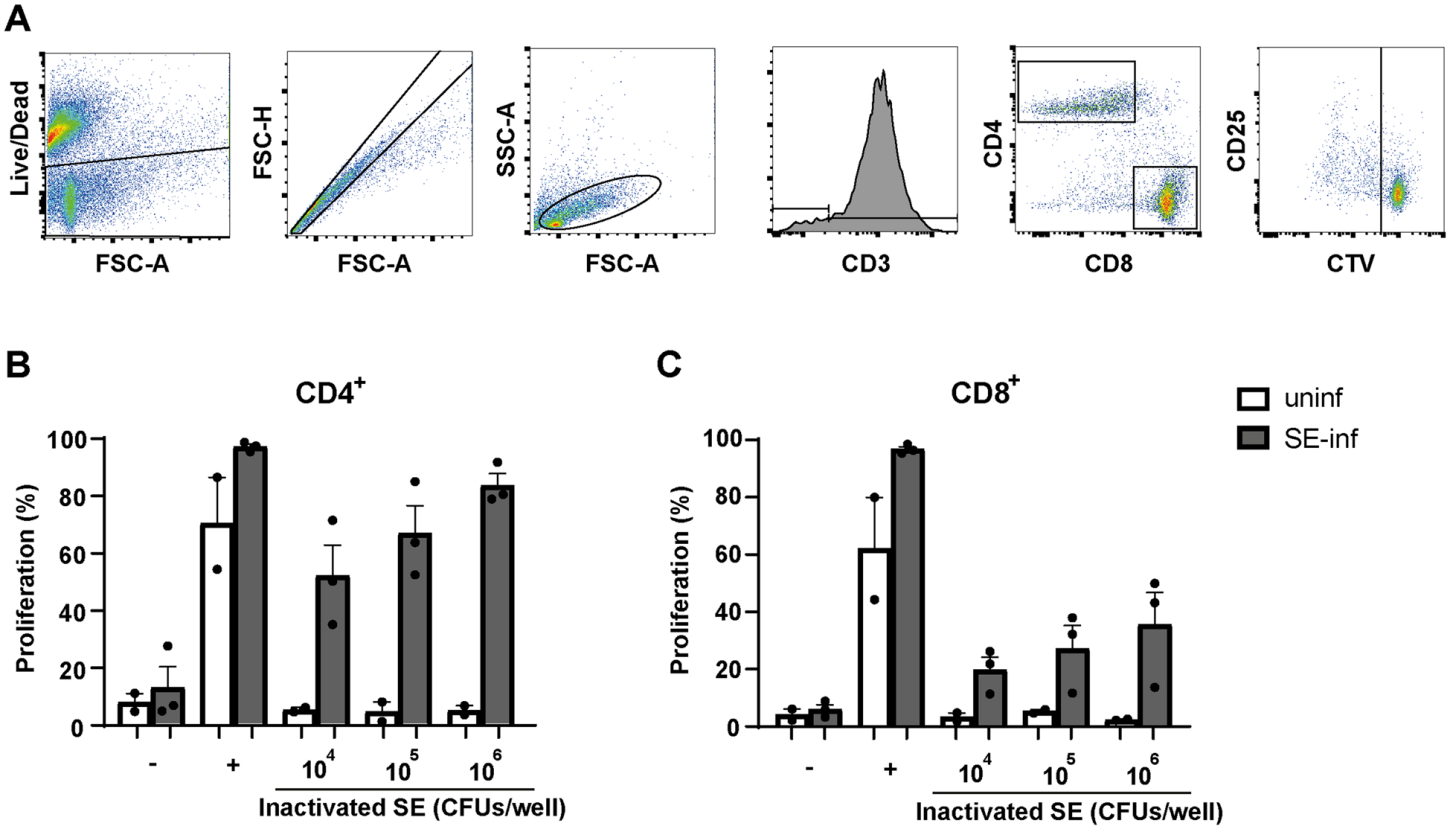
**Ex vivo proliferation of SE-induced splenic CD4**^**+**^** and CD8**^**+**^** T cells.A** The gating strategy shows the consecutive selection for viable cells (Live/Dead marker-negative), single cells (FSC-A vs FSC-H), lymphocytes (FSC-A vs SSC-A) and CD3^+^ T cells. T cells were subdivided into CD4^+^ and CD8^+^ T cells. The final gating step selects on T cell subsets which have divided at least once based on dilution of the cell proliferation dye CellTrace Violet (CTV). **B** The percentage of cells that have proliferated is shown for splenic CD4^+^ and **C** CD8^+^ T cells after 4 days of stimulation with different doses of formaldehyde-inactivated SE expressed in CFU/well, none stimulated controls (−), or after stimulation with anti-CD3, anti-CD28 and recombinant chicken IL-2 ( +). All splenocyte samples were stimulated and measured in triplicate for each of the conditions. Mean + SEM is shown; *n* = 2 for uninfected (uninf) and *n* = 3 for SE-infected (SE-inf) chickens.

SE-induced proliferation of T cells, isolated from spleen at 21 dpi, was determined ex vivo (Figure 7A). Increased proliferation of SE-induced CD4^+^ as well as CD8^+^ T cells isolated from SE-infected chickens was observed. This proliferation was antigen dose-dependent, whereas T cells from uninfected chickens did not proliferate upon exposure to inactivated SE (Figure 7B, C).

### High SE-specific antibody responses were found in all SE-infected chickens after 3 weeks of infection

**Figure 8:**
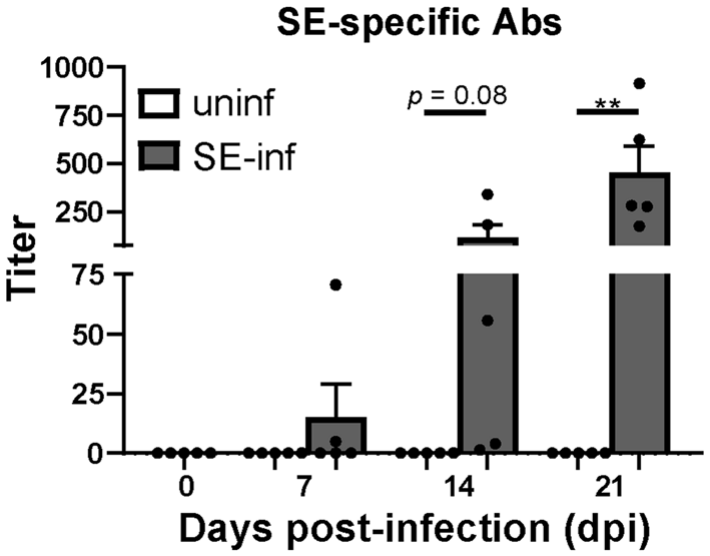
**Serum antibody titers in broiler chickens as a response to SE infection.** Titers of SE-specific antibodies in sera of uninfected (uninf) and SE-infected (SE-inf) chickens in course of time. Mean + SEM per treatment and time point is shown (*n* = 5) and statistical significance is indicated as ** *p* < 0.01.

The presence of SE-specific antibodies was determined in serum before and after infection in uninfected and SE-infected chickens. In SE-infected chickens, SE-specific antibodies were first detected at 7 dpi, when two out of five chickens showed low antibody titers, that increased in course of time (Figure 8). At 14 dpi SE-specific antibodies were observed in all SE-infected chickens although two chickens showed only low titers. At 21 dpi all SE-infected chickens showed high SE-specific antibody responses. SE-specific antibodies were not found in sera of the uninfected chickens (Figure 8).

## Discussion

In the current study we aimed to provide a detailed analysis of SE-related innate and adaptive immune responses in young broiler chickens up to 4 weeks of age, to better understand how the immune response contributes to the elimination of infection in course of time. For this purpose, the presence and function of NK cells, various types of APCs and T cells in ileum, as the present infection site and in spleen, as indication of systemic dissemination of SE, were investigated, as well as SE-specific antibody responses in serum, another systemic dissemination indicator. Seven-day-old broiler chickens were successfully infected with SE as was demonstrated by the detection of SE-induced T cell proliferation and SE-specific antibodies from 2–3 weeks after infection. Presence of SE was detected in ileum only at day 7 post-infection and in spleen from day 7 onwards and most likely, the number of bacteria in these tissues were below the detection limit during the first week post-infection as observed previously [43]. In the first week post-infection, significant increases in numbers of intraepithelial cytotoxic T cells and splenic macrophages were observed in SE-infected compared to uninfected chickens. This was paralleled by a significant increase in NK cell activation in the IEL population and spleen as well as DC activation in the spleen in SE-infected compared to uninfected chickens. At 21 dpi, antibody titers in serum were significantly increased in SE-infected compared to uninfected chickens. These immune responses were paralleled by a reduction in SE counts.

Although presence of SE was demonstrated in ileum and spleen of infected chickens, SE infection did not affect growth nor induced severe disease symptoms. This observation was similar to previous studies in young broiler chickens that were infected at 7 or 9 to 11 days of age [40, 60] and in layer chickens that were infected during adult life [20, 43]. The absence of severe disease symptoms is related to the SE-dose, which was chosen to avoid welfare issues in the chickens. Although SE has been detected in the small intestine [61, 62], other studies reported the presence of SE for a longer period in the caecum [40, 41]. This suggests that SE may prefer colonization in the caecum rather than the ileum.

The enhanced activation of intraepithelial and splenic NK cells upon SE infection, represented by enhanced CD107 expression and IFNγ production, is in agreement with other studies in chickens showing upregulated mRNA levels of intestinal IFNγ [20] and cytotoxicity-related NK cell genes [19] in young chickens. Our observations are also supported by studies in humans and mice, which reported increased cytotoxicity [23], IFNγ production [25] and CD107 expression [24] of intestinal and systemic NK cells after *Salmonella enterica* serotype Typhimurium infection. The enhanced NK cell activation paralleled numerically increased intraepithelial IL-2Rα^+^ and 20E5^+^ NK cells. Although we did not observe a distinct population of IL-2Rα^+^ 20E5^+^ cells, we cannot exclude the possibility that these cells exist at a very low frequency. Due to incompatibility of available reagents, we were not able to determine CD107 and IFNγ expression within the IL-2Rα^+^ and 20E5^+^ NK cell subsets. For that reason, we sorted IL-2Rα^+^ and 20E5^+^ NK cells to perform RT-qPCR and both NK cell subsets showed mRNA levels of *NFIL3*, *IL-7Rα* and *PRF1* genes albeit to different degrees, suggesting that both may be implicated in cytokine production [50–52] as well as cytotoxic activity [52, 55] in response to SE infection. The observation that both NK cell subsets are involved in cytotoxicity is confirmed by previous studies in chickens, in which both NK cell subsets in the IEL population and spleen showed CD107 expression [12] and intraepithelial IL-2Rα^+^ NK cells exerted cytotoxicity [11]. In humans, the peripheral IL-2Rα^+^ NK cell population expanded and increased their cytotoxic activity after Toll-like receptor (TLR) stimulation [63]. As SE might activate NK cells directly through TLRs [63–65], more intraepithelial NK cells as well as enhanced activation, such as cytotoxicity and IFNγ production, may increase the resistance of chickens against SE infection. IFNγ has been reported to activate macrophages resulting in improved clearance of engulfed bacteria [66] and enhanced antigen presentation by APCs inducing T cell responses [67]. Although we were unable to demonstrate a direct relation between NK cell activation and SE counts in the first week post-infection, we hypothesize that NK cells play an important role in the resistance against SE infection. This can be either via direct killing of infected cells or indirectly by influencing other innate and adaptive immune cells via the production of IFNγ.

The systemic spread of SE infection as observed, coincided with a significantly increased presence of CD11^+^ MRC1LB^+^ macrophages in the spleen at 7 dpi when bacterial counts were highest. Previous studies have shown that MRC1LB^+^ macrophages are largely present in peri-ellipsoid lymphocyte sheaths of the spleen [68], and have a role in clearing blood-borne bacteria in chickens [69], equivalent to that of the marginal zone macrophages in mammals [70]. Therefore, these macrophages are suggested to be involved in clearing the SE from 7 dpi onwards. Expression levels of MHC-II found on MRC1LB^+^ macrophages were higher than those found on the DC subpopulations, which was a surprising finding. In a recent publication, similarly high MHC-II expression levels were observed for chicken splenic MRC1LB^+^ macrophages but this level of MHC-II expression was also found on DCs [71]. The FSC^high^ DCs were numerically higher in presence and showed significant increased expression of CHIR-AB1 and MHC-II at 7 dpi in SE-infected chickens compared to uninfected chickens, indicating a role in antigen presentation. The FSC^low^ DCs did not increase in numbers but showed a significant increased expression of the activation markers CHIR-AB1, CD40 and CD80 in SE-infected chickens. The two DC subsets were highly similar, and might comprise DCs at different stages of maturation with the FSC^low^ subset being more mature based on the expression of CD40, CD80 and MHCII [31, 58, 72]. These results suggest that the increased presence of macrophages clear bacteria initially and the increased activation of DC subsets contribute to antigen presentation to stimulate the adaptive immune responses, all together resulting in further reduction of SE in infected chickens.

Whereas the APC subsets are likely to contribute to the clearance of the bacteria, it has also been suggested that they may worsen the impact of infection by acting as a carrier for *Salmonellae* [32] and contribute to systemic dissemination [37], since this bacterium is able to survive intracellularly in chicken macrophages [35, 36] and DCs [72]. It would be interesting for future studies to determine whether SE can be detected by qPCR in splenic APCs. The ability of *Salmonellae* to inhibit activation of APCs might explain why NK cells showed earlier activation than APCs and the high presence of SE found at 7 dpi in our study. Other studies have demonstrated that *Salmonella*-infected APCs secrete IL-12/IL-18 resulting in enhanced expression of the early activation marker IL-2Rα^+^ on NK cells, thereby inducing their activation by increased cytotoxicity and IFNγ production [24]. This IFNγ production can subsequently stimulate additional macrophages to clear phagocytosed bacteria [73] or impair intracellular survival by direct killing of infected-macrophages [24], which might be involved in the reduction of SE towards and after 7 dpi observed in our study.

T cell presence, SE-induced T cell proliferation and SE specific antibodies were addressed as well. All infected chickens in our study had circulating antibodies specific for SE after 3 weeks, which was in agreement with other studies [40, 41]. The observed T cell responses are similar to increased numbers of intestinal and splenic γδ and cytotoxic αβ T cells in response to SE early and 3 weeks after infection [20, 26, 27], as well as to increased CD8αα^+^ γδ T cell numbers [22, 74] in prior studies in chickens. The significant increase in cytotoxic CD8αα^+^ T cell numbers, however, has not been shown before in chickens in response to SE infection. The more innate-like nature of γδ T cell responses early after infection have been recognized as well as the antigen-specific responses of cytotoxic CD8^+^ T cells approximately 2 weeks after infection, whereas the functional difference between CD8αα and CD8αβ expression is less clear [75–78]. Although expression of CD107 and IFNγ production by intraepithelial and splenic γδ T cells, cytotoxic CD8^+^ T cells, and splenic helper CD4^+^ T cells did not significantly differ between uninfected and SE-infected chickens, proliferation of SE-induced splenic T cells of SE-infected chickens ex vivo was observed 3 weeks after infection and not in uninfected chickens. Although the effect of the initial increased presence of T cells on the numbers of SE in the first week post-infection could not be determined, the SE-specific T cells and antibodies in course of infection are suggested to reduce the number of SE.

In conclusion, this study shows that *Salmonella enterica* serotype Enteritidis infection in young broiler chickens firstly induces local and systemic activation of NK cells (1, 3, 7 dpi) as well as presence of intraepithelial T cells (1 dpi), followed by increased presence of macrophages and activation of DCs (7 dpi). Subsequently, proliferation of T cells in the spleen and antibody responses in serum (21 dpi) are induced, all together paralleled by a reduction in SE counts. These insights in understanding the role of NK cell and APC subsets and responses of adaptive immune cells upon SE infection will aid in developing immune-modulation strategies to stimulate innate cells. The potential strengthening of immune responsiveness by vaccines or feed strategies during early life may increase resistance and may prevent SE infection and colonization in young broiler chickens.

## Supplementary Information

**Additional file 1. Gating strategy of IELs and splenic lymphocytes in broiler chickens.** Gating strategy included consecutive selection for lymphocytes (FSC-A vs SSC-A), singlets (FSC-A vs FSC-H) and viable cells (Live/Dead marker-negative) followed by selection of NK and T cell subsets in ileum and spleen. Furthermore, activation of NK and T cells was analyzed by surface expression of CD107 and intracellular expression of IFNγ. Conjugate controls are shown for IELs and splenic lymphocytes.

**Additional file 2. Effect of SE infection on numbers of splenic NK cells in broiler chickens.A** Numbers (cells/mg) of splenic IL-2Rα^+^ and **B** 20E5^+^ NK cells per mg spleen in uninfected (uninf) and SE-infected (SE-inf) chickens in the course of time. **C** Gene expression levels of NK cell lineage marker (NFIL3), IL-7Rα and perforin 1 (PRF1) by RT-qPCR in sorted IL-2Rα^+^ and 20E5^+^ NK cell subsets. Mean + SEM per treatment and time point is shown (*n* = 5), for uninfected chickens at 7 dpi *n* = 4 and for gene expression levels *n* = 1.

**Additional file 3. Staining and sorting controls associated with Figure **4**. A** The staining controls for the gating strategy are shown. The left panel depicts splenocytes without the viability dye. The middle and right panels show splenocytes that are gated according to Figure 4A, but without the primary antibodies that bind MRC1LB and CD11, respectively. **B** The graphs show the gating strategy and purity of a representative sample of splenocytes that was sorted into CD11^+^ MRC1LB^+^, CD11^+^ MRC1LB^−^ FSC^low^ and CD11^+^ MRC1LB^−^ FSC^high^ subpopulations. The splenocytes that are gated as CD11^+^ MRC1LB^−^ in the upper panels are shown in the lower panels to visualize their FSC-A vs SSC-A pattern. **C** The absolute numbers of sorted APC subpopulations are shown.

**Additional file 4. Phenotypic characterization of splenic APCs upon SE infection.A-B** The presence (%) and **C-D** numbers (cells/mg spleen) of FSC^low^ DCs and and FSC^high^ DCs in uninfected (uninf) and SE-infected (SE-inf) chickens were assessed over time. Mean + SEM per treatment and time point is shown (*n* = 5), for uninfected chickens at 0 dpi *n* = 3 and at 7 dpi *n* = 4. Statistical significance is indicated as ** *p* < 0.01.

**Additional file 5. The gating strategy used to determine the activation status of the APC subsets as depicted in Figure **5**.** The three identified splenic APC subsets **A** macrophages, **B** FSC^low^ DCs and **C** FSC^high^ DCs were assessed for CHIR-AB1, CD40, CD80 and MHC-II. For CHIR-AB1, CD40 and CD80, the cells expressing the respective markers were selected and expressed as a percentage. The expression of MHC-II by each subset was expressed as the geometric mean fluorescent intensity (gMFI).

**Additional file 6. Numbers of intraepithelial and splenic γδ T cells and cytotoxic T cells expressing either CD8αα or CD8αβ in broiler chickens upon SE infection.A** Numbers (cells/mg) of intraepithelial CD8αα^+^ γδ T cells, **B** CD8αβ^+^ γδ T cells, **C** cytotoxic CD8αα^+^ T cells and **D** CD8αβ^+^ T cells per mg ileum in uninfected (uninf) and SE-infected (SE-inf) chickens in the course of time. **E** Numbers (cells/mg) of splenic CD8αα^+^ γδ T cells, **F** CD8αβ^+^ γδ T cells, **G** cytotoxic CD8αα^+^ T cells and **H** CD8αβ^+^ T cells per mg spleen in uninfected and SE-infected chickens. Mean + SEM per treatment and time point is shown (*n* = 5), for uninfected chickens at 1 dpi in the IELs and spleen *n* = 4 due to numbers of events acquired in the gate of interest were < 100, and at 7 dpi in spleen *n* = 4. Statistical significance is indicated as * *p* < 0.05, ** *p* < 0.01. *** *p* < 0.001.

**Additional file 7. Numbers of CD4**^+^**T cells in the spleen of broiler chickens upon SE infection.** Numbers (cells/mg) of splenic CD4^+^ αβ T cells per mg spleen in uninfected (uninf) and SE-infected (SE-inf) chickens in the course of time. Mean + SEM per treatment and time point is shown (*n* = 5), for uninfected chickens at 7 dpi *n* = 4.

**Additional file 8. T cell activation in the IEL population and spleen of broiler chickens upon SE infection.A** Percentages of intraepithelial CD8^+^ T cells expressing CD107 (including both γδ and αβ T cells) in uninfected (uninf) and SE-infected (SE-inf) chickens in the course of time. **B** Percentages of splenic CD8^+^ T cells expressing CD107 (including both γδ and αβ T cells), **C** CD8^+^ γδ T cells expressing IFNγ, **D** CD4^+^ αβ T cells expressing IFNγ and **E** CD8^+^ αβ T cells expressing IFNγ in uninfected (uninf) and SE-infected (SE-inf) chickens over time. Mean + SEM per treatment and time point is shown (*n* = 5), for uninfected chickens at 7 dpi in spleen *n* = 4 and at 1 and 3 dpi in the IELs percentages were not determined (n.d.) due to numbers of events acquired in the gate of interest were < 100.

## Data Availability

All data generated or analyzed during this study are included in this published article [and its additional information files].

## Abbreviations

SE: *Salmonella enterica* Serotype Enteritidis
NK cell: Natural killer cell
IELs: Intraepithelial lymphocytes
TCR: T cell receptor
DC: Dendritic cell
APC: Antigen-presenting cell
MRC1LB: Mannose receptor C-type 1-like B
dpi: Day(s) post-infection
ED: Embryonic day
CFU: Colony-forming unit
LOD: Limit of detection
RT: Room temperature
gMFI: Geometric mean fluorescent intensity
FACS: Fluorescence-activated cell sorting
CTV: CellTrace Violet
uninf: Uninfected
SE-inf: SE-infected
