# Supplementary figures and images for: A detailed analysis of innate and adaptive immune responsiveness upon infection with Salmonella enterica serotype Enteritidis in young broiler chickens
Source: Vet Res. 2021 Aug 17;52:109. doi: 10.1186/s13567-021-00978-y (PMC8369617; doi:10.1186/s13567-021-00978-y)

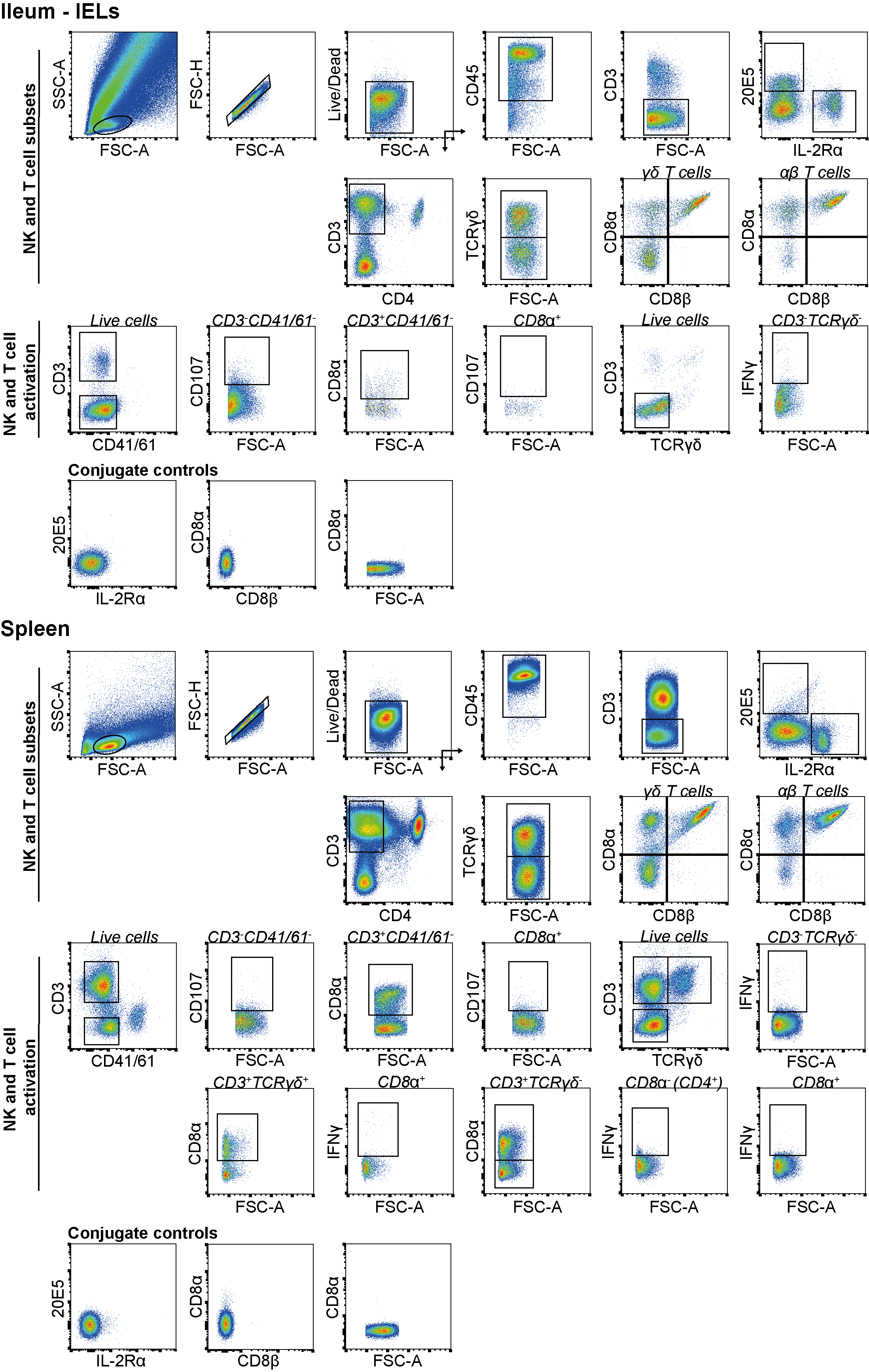

Supplement: Supplementary file 1 — Additional file 1. Gating strategy of IELs and splenic lymphocytes in broiler chickens. Gating strategy included consecutive selection for lymphocytes (FSC-A vs SSC-A), singlets (FSC-A vs FSC-H) and viable cells (Live/Dead marker-negative) followed by selection of NK and T cell subsets in ileum and spleen. Furthermore, activation of NK and T cells was analyzed by surface expression of CD107 and intracellular expression of IFNγ. Conjugate controls are shown for IELs and splenic lymphocytes. [file 13567_2021_978_MOESM1_ESM.tif]

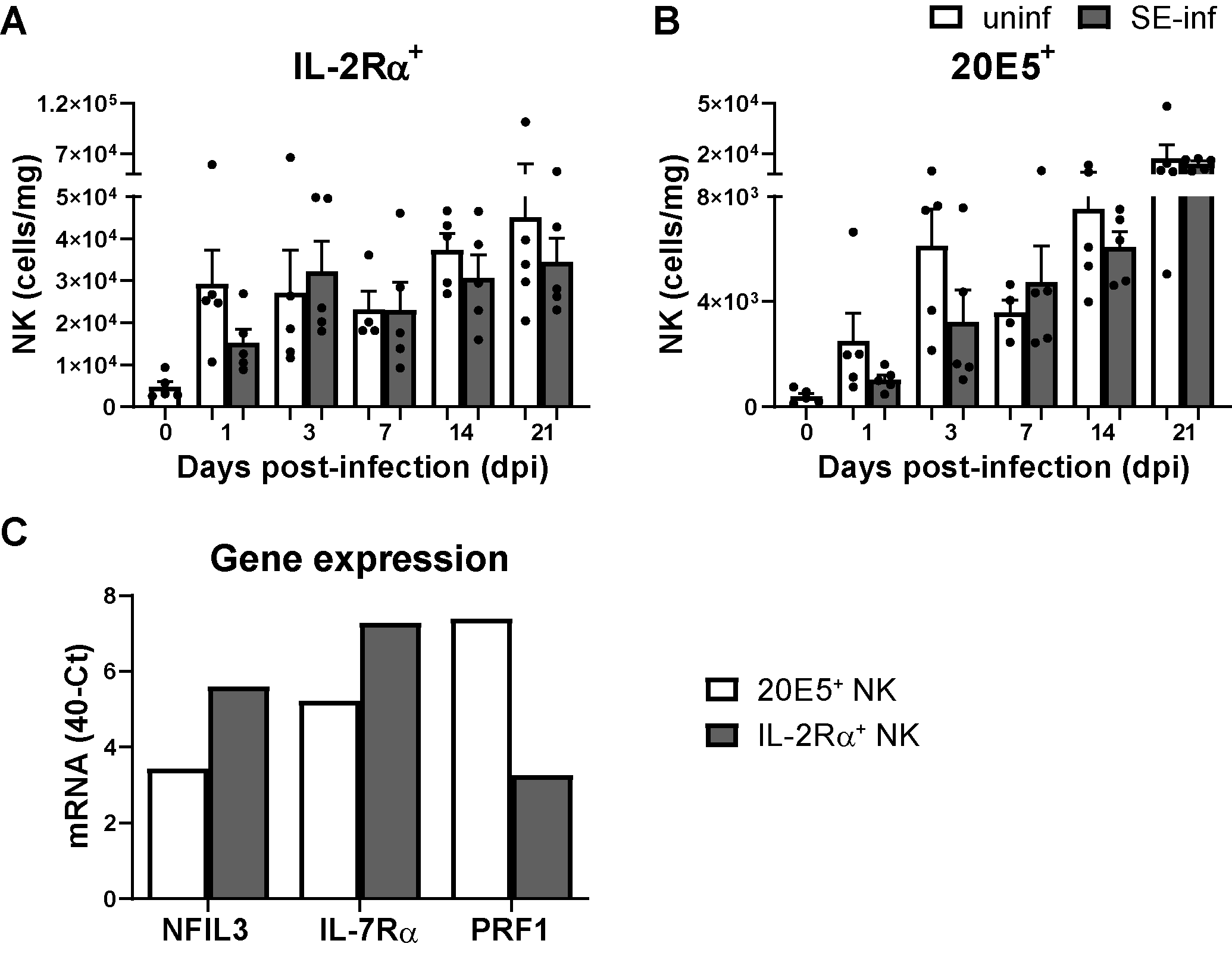

Supplement: Supplementary file 2 — Additional file 2. Effect of SE infection on numbers of splenic NK cells in broiler chickens.A Numbers (cells/mg) of splenic IL-2Rα+ and B 20E5+ NK cells per mg spleen in uninfected (uninf) and SE-infected (SE-inf) chickens in the course of time. C Gene expression levels of NK cell lineage marker (NFIL3), IL-7Rα and perforin 1 (PRF1) by RT-qPCR in sorted IL-2Rα+ and 20E5+ NK cell subsets. Mean + SEM per treatment and time point is shown (n = 5), for uninfected chickens at 7 dpi n = 4 and for gene expression levels n = 1. [file 13567_2021_978_MOESM2_ESM.tif]

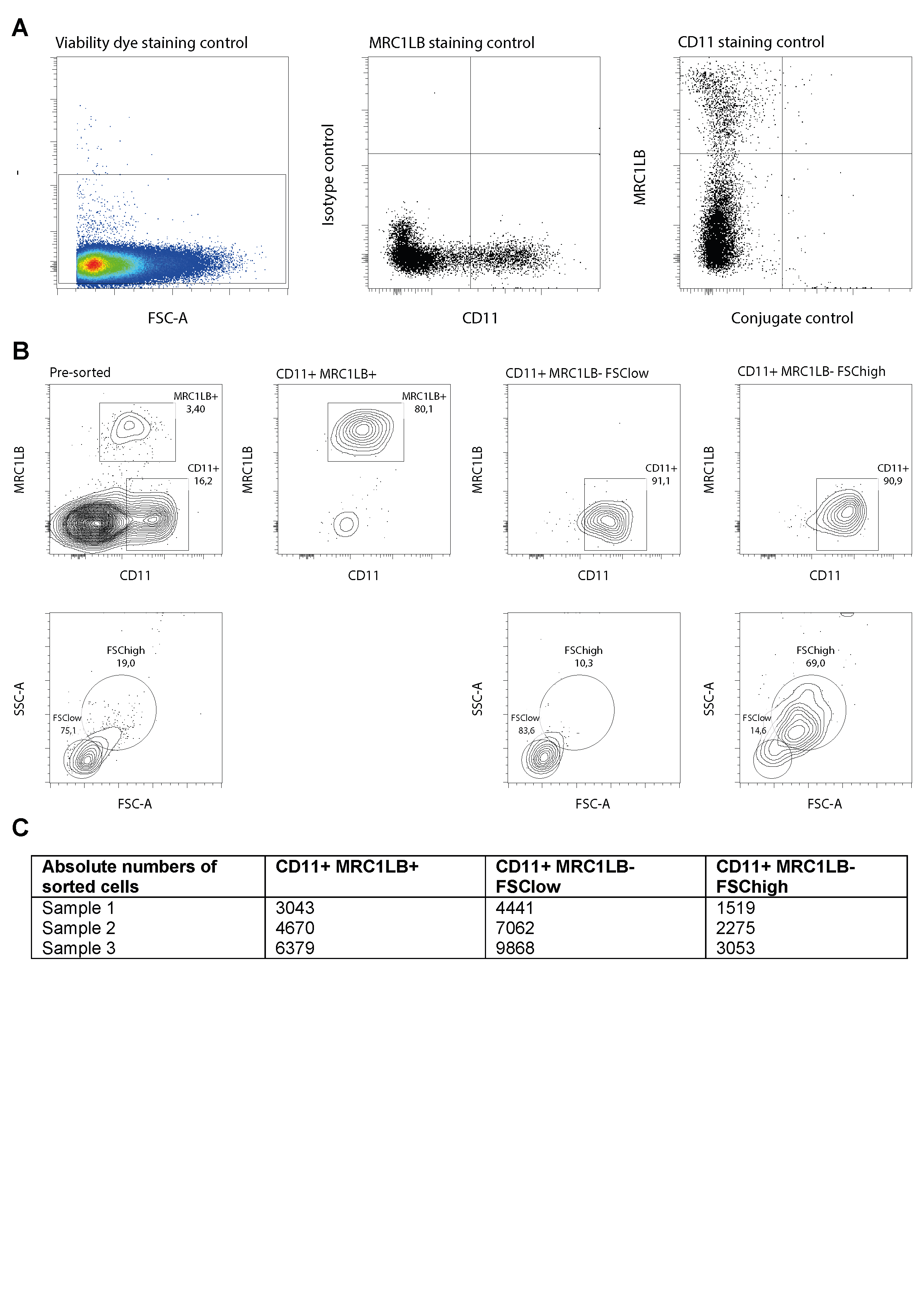

Supplement: Supplementary file 3 — Additional file 3. Staining and sorting controls associated with Figure 4. A The staining controls for the gating strategy are shown. The left panel depicts splenocytes without the viability dye. The middle and right panels show splenocytes that are gated according to Figure 4A, but without the primary antibodies that bind MRC1LB and CD11, respectively. B The graphs show the gating strategy and purity of a representative sample of splenocytes that was sorted into CD11+ MRC1LB+, CD11+ MRC1LB− FSClow and CD11+ MRC1LB− FSChigh subpopulations. The splenocytes that are gated as CD11+ MRC1LB− in the upper panels are shown in the lower panels to visualize their FSC-A vs SSC-A pattern. C The absolute numbers of sorted APC subpopulations are shown. [file 13567_2021_978_MOESM3_ESM.tif]

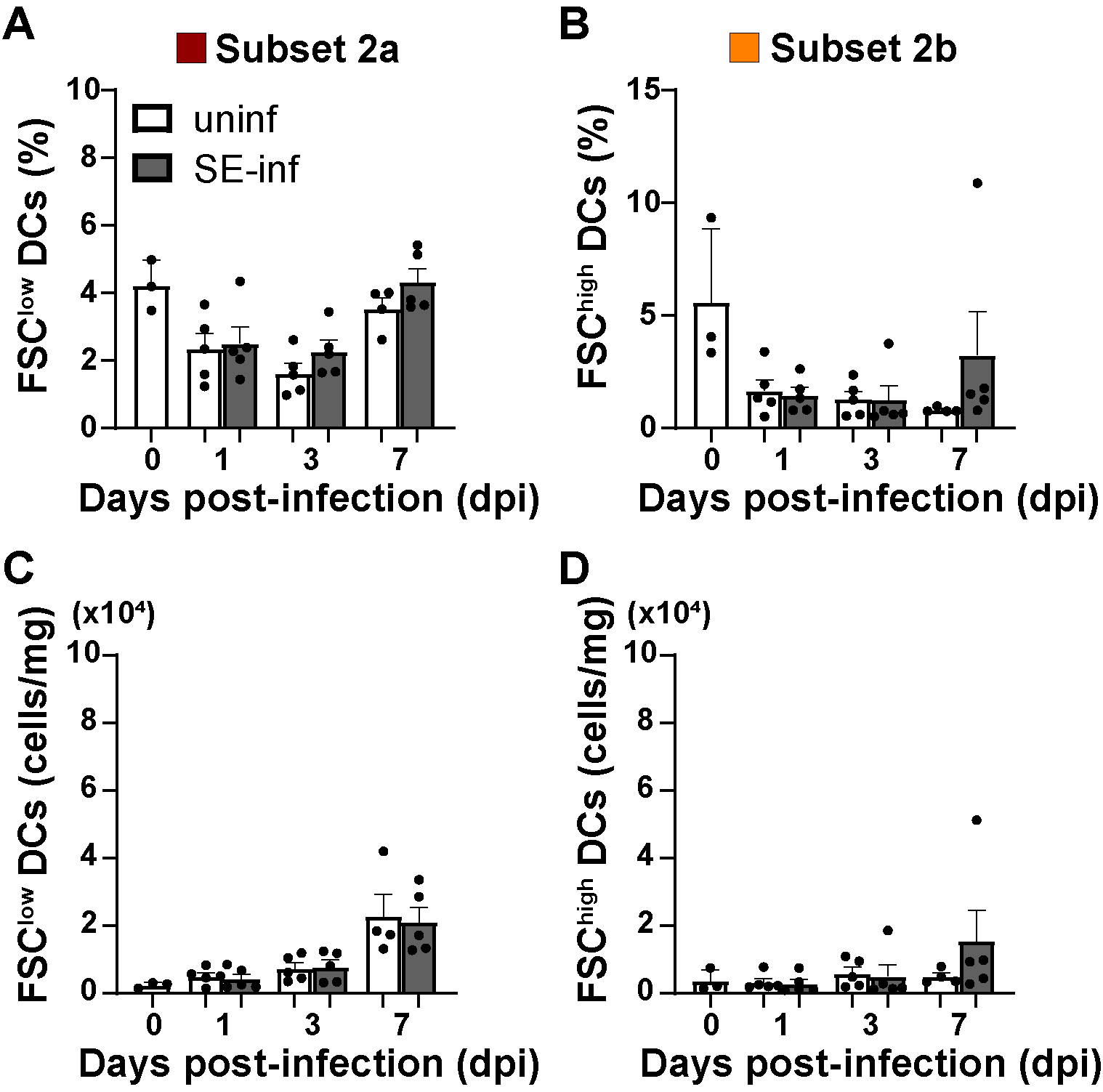

Supplement: Supplementary file 4 — Additional file 4. Phenotypic characterization of splenic APCs upon SE infection.A-B The presence (%) and C-D numbers (cells/mg spleen) of FSClow DCs and and FSChigh DCs in uninfected (uninf) and SE-infected (SE-inf) chickens were assessed over time. Mean + SEM per treatment and time point is shown (n = 5), for uninfected chickens at 0 dpi n = 3 and at 7 dpi n = 4. Statistical significance is indicated as ** p < 0.01. [file 13567_2021_978_MOESM4_ESM.tif]

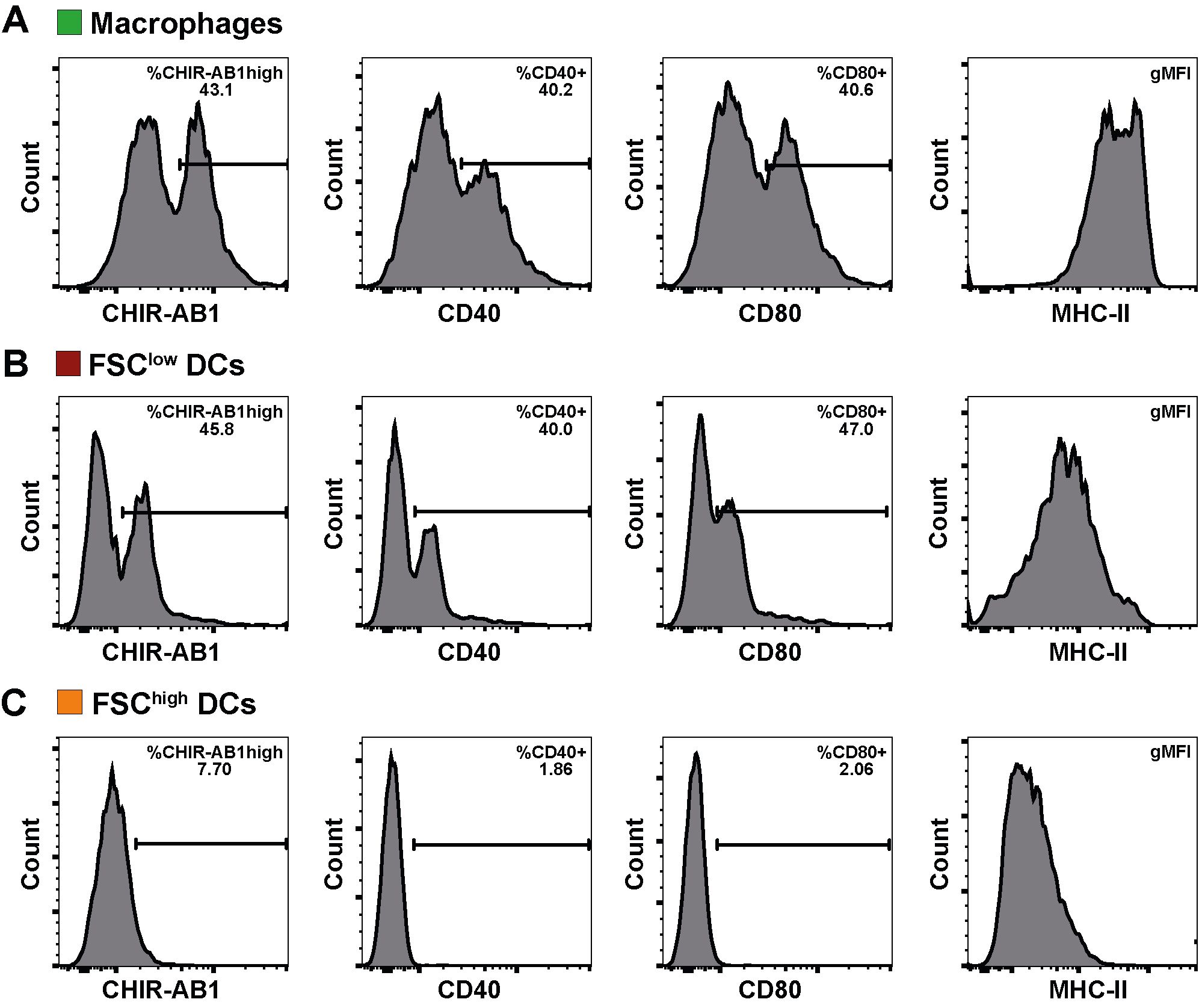

Supplement: Supplementary file 5 — Additional file 5. The gating strategy used to determine the activation status of the APC subsets as depicted in Figure 5. The three identified splenic APC subsets A macrophages, B FSClow DCs and C FSChigh DCs were assessed for CHIR-AB1, CD40, CD80 and MHC-II. For CHIR-AB1, CD40 and CD80, the cells expressing the respective markers were selected and expressed as a percentage. The expression of MHC-II by each subset was expressed as the geometric mean fluorescent intensity (gMFI). [file 13567_2021_978_MOESM5_ESM.tif]

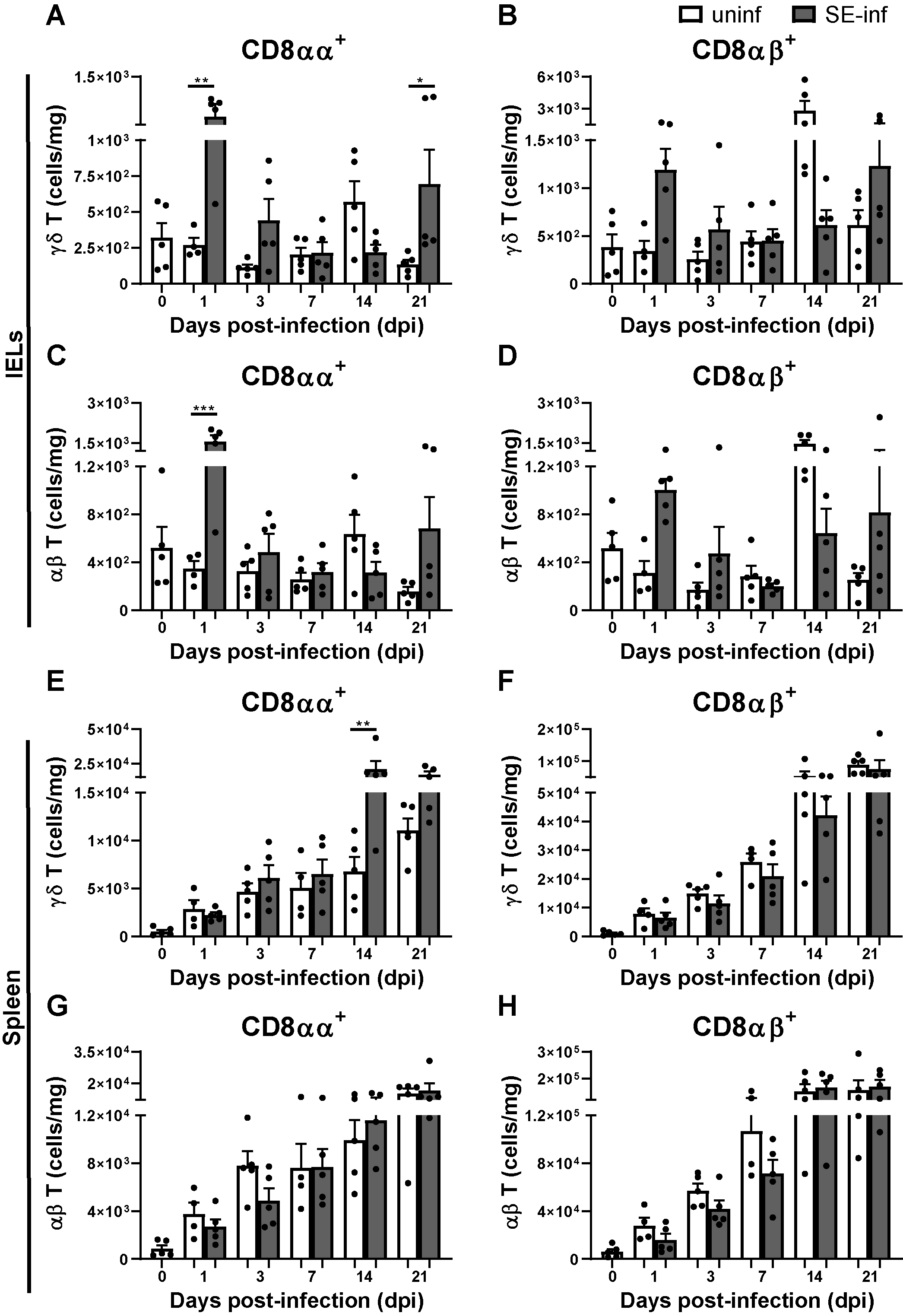

Supplement: Supplementary file 6 — Additional file 6. Numbers of intraepithelial and splenic γδ T cells and cytotoxic T cells expressing either CD8αα or CD8αβ in broiler chickens upon SE infection.A Numbers (cells/mg) of intraepithelial CD8αα+ γδ T cells, B CD8αβ+ γδ T cells, C cytotoxic CD8αα+ T cells and D CD8αβ+ T cells per mg ileum in uninfected (uninf) and SE-infected (SE-inf) chickens in the course of time. E Numbers (cells/mg) of splenic CD8αα+ γδ T cells, F CD8αβ+ γδ T cells, G cytotoxic CD8αα+ T cells and H CD8αβ+ T cells per mg spleen in uninfected and SE-infected chickens. Mean + SEM per treatment and time point is shown (n = 5), for uninfected chickens at 1 dpi in the IELs and spleen n = 4 due to numbers of events acquired in the gate of interest were < 100, and at 7 dpi in spleen n = 4. Statistical significance is indicated as * p < 0.05, ** p < 0.01. *** p < 0.001. [file 13567_2021_978_MOESM6_ESM.tif]

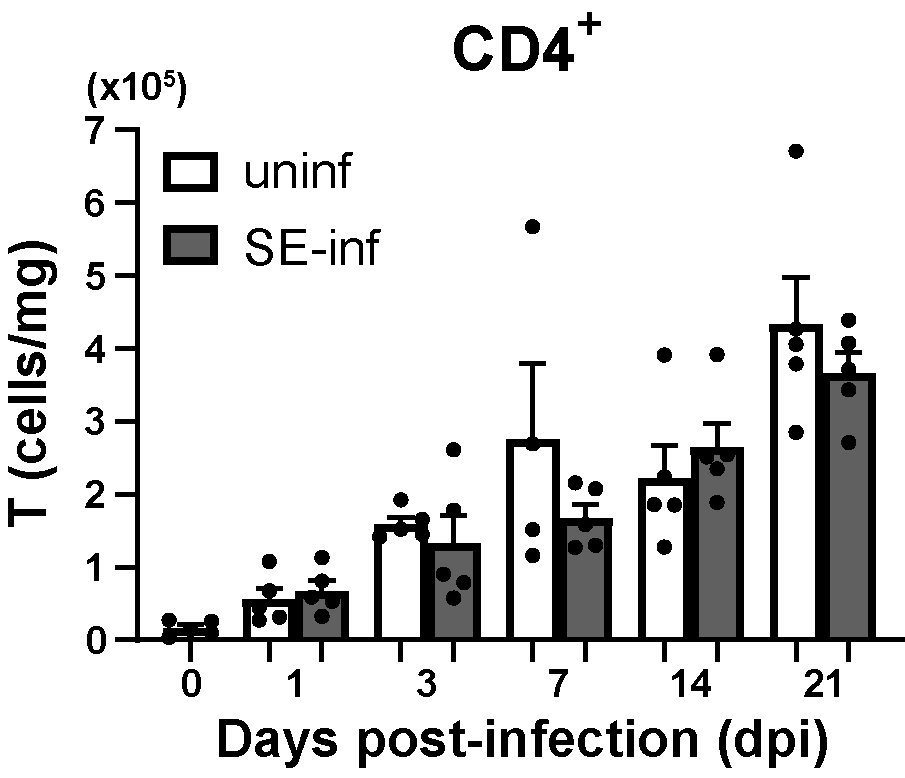

Supplement: Supplementary file 7 — Additional file 7. Numbers of CD4+T cells in the spleen of broiler chickens upon SE infection. Numbers (cells/mg) of splenic CD4+ αβ T cells per mg spleen in uninfected (uninf) and SE-infected (SE-inf) chickens in the course of time. Mean + SEM per treatment and time point is shown (n = 5), for uninfected chickens at 7 dpi n = 4. [file 13567_2021_978_MOESM7_ESM.tif]

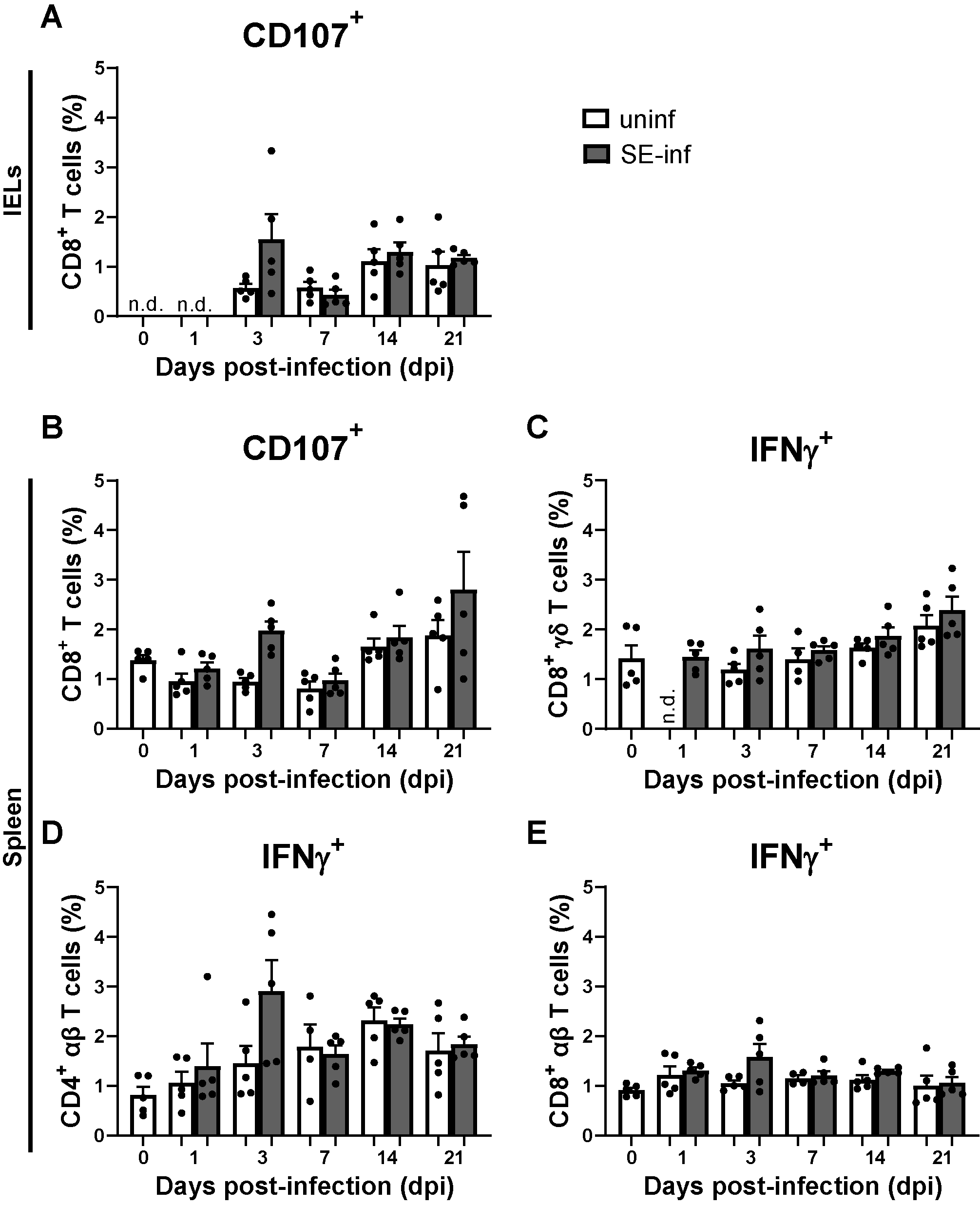

Supplement: Supplementary file 8 — Additional file 8. T cell activation in the IEL population and spleen of broiler chickens upon SE infection.A Percentages of intraepithelial CD8+ T cells expressing CD107 (including both γδ and αβ T cells) in uninfected (uninf) and SE-infected (SE-inf) chickens in the course of time. B Percentages of splenic CD8+ T cells expressing CD107 (including both γδ and αβ T cells), C CD8+ γδ T cells expressing IFNγ, D CD4+ αβ T cells expressing IFNγ and E CD8+ αβ T cells expressing IFNγ in uninfected (uninf) and SE-infected (SE-inf) chickens over time. Mean + SEM per treatment and time point is shown (n = 5), for uninfected chickens at 7 dpi in spleen n = 4 and at 1 and 3 dpi in the IELs percentages were not determined (n.d.) due to numbers of events acquired in the gate of interest were < 100. [file 13567_2021_978_MOESM8_ESM.tif]
